# TCP4-dependent induction of *CONSTANS* transcription requires GIGANTEA in photoperiodic flowering in *Arabidopsis*

**DOI:** 10.1371/journal.pgen.1006856

**Published:** 2017-06-19

**Authors:** Akane Kubota, Shogo Ito, Jae Sung Shim, Richard S. Johnson, Yong Hun Song, Ghislain Breton, Greg S. Goralogia, Michael S. Kwon, Dianne Laboy Cintrón, Tomotsugu Koyama, Masaru Ohme-Takagi, Jose L. Pruneda-Paz, Steve A. Kay, Michael J. MacCoss, Takato Imaizumi

**Affiliations:** 1 Department of Biology, University of Washington, Seattle, Washington, United States of America; 2 Department of Genome Sciences, University of Washington, Seattle, Washington, United States of America; 3 Department of Life Sciences, Ajou University, Suwon, Korea; 4 Section of Cell and Developmental Biology, Division of Biological Sciences, University of California at San Diego, La Jolla, California, United States of America; 5 Bioorganic Research Center, Suntory Foundation for Life Sciences, Kyoto, Japan; 6 Graduate School of Science and Engineering, Saitama University, Saitama, Japan; 7 Keck School of Medicine, University of Southern California, Los Angeles, California, United States of America; University of California Berkeley, UNITED STATES

## Abstract

Photoperiod is one of the most reliable environmental cues for plants to regulate flowering timing. In *Arabidopsis thaliana*, CONSTANS (CO) transcription factor plays a central role in regulating photoperiodic flowering. In contrast to posttranslational regulation of CO protein, still little was known about *CO* transcriptional regulation. Here we show that the CINCINNATA (CIN) clade of class II TEOSINTE BRANCHED 1/ CYCLOIDEA/ PROLIFERATING CELL NUCLEAR ANTIGEN FACTOR (TCP) proteins act as *CO* activators. Our yeast one-hybrid analysis revealed that class II CIN-TCPs, including TCP4, bind to the *CO* promoter. TCP4 induces *CO* expression around dusk by directly associating with the *CO* promoter *in vivo*. In addition, TCP4 binds to another flowering regulator, GIGANTEA (GI), in the nucleus, and induces *CO* expression in a *GI*-dependent manner. The physical association of TCP4 with the *CO* promoter was reduced in the *gi* mutant, suggesting that GI may enhance the DNA-binding ability of TCP4. Our tandem affinity purification coupled with mass spectrometry (TAP-MS) analysis identified all class II CIN-TCPs as the components of the *in vivo* TCP4 complex, and the *gi* mutant did not alter the composition of the TCP4 complex. Taken together, our results demonstrate a novel function of CIN-TCPs as photoperiodic flowering regulators, which may contribute to coordinating plant development with flowering regulation.

## Author summary

For plant adaptation to seasonal environments, a crucial developmental event is flowering, as proper timing of flowering affects reproductive success. Although plants monitor various environmental parameters to optimize this timing, photoperiod information is important for plants to regulate seasonal flowering time, because changes in photoperiod occur in a predictable manner throughout the year. The model plant *Arabidopsis thaliana* responds to photoperiodic changes and flowers under long-day conditions. Based on genetic analyses using mutants defective in the photoperiodic flowering response, we learned that the transcription factor referred to as CONSTANS (CO) plays a central role in regulating the timing of flowering by directly controlling the expression of florigen (flowering-inducing substrate) gene. Long-day afternoon expression of *CO* is critical for this regulation; however, we had limited knowledge of *CO* transcriptional regulation. Here we identified that a group of plant-specific transcription factors belonging to the *TCP* gene family function as novel *CO* transcriptional activators. We demonstrated that TCP transcription factors regulate *CO* transcription together with known regulators of *CO*. Our results imply that plants utilize multiple transcription factors to precisely coordinate the expression of the key regulator gene, *CO*, which will directly affect flowering time.

## Introduction

The transition from vegetative to reproductive growth phases at the most appropriate season is crucial for plant reproductive success. One of the most influential environmental cues that induces seasonal response is day length (= photoperiod). In the model plant *Arabidopsis thaliana*, the growth-phase transition, which is observed as flowering, is promoted in long days (LD). Flowering is induced by the expression of *FLOWERING LOCUS T* (*FT*), which is induced specifically at dusk during LD [1–3]. *FT* encodes a florigen synthesized in leaf phloem companion cells and transported to the shoot apical meristem, where FT protein interacts with the bZIP transcription factor FD and 14-3-3 protein to start orchestrating the expression of the floral identity genes [4–6]. CONSTANS (CO), a transcriptional activator containing two B-box and CO, CO-Like, TOC1 (CCT) domains, is expressed in leaf phloem companion cells and directly activates *FT* transcription [7–11]. It is widely accepted that day length information is mainly integrated into this pathway by controlling CO functions in *Arabidopsis*.

Transcriptional regulation of *CO* is the first crucial step to properly induce photoperiodic flowering [1, 2, 12, 13]. Currently, two groups of transcription factors are known as direct regulators of *CO* expression; CYCLING DOF FACTOR (CDF) of the DNA binding with One Finger (DOF) family and FLOWERING BHLH (FBH) of the basic Helix-Loop-Helix (bHLH) family [14–16]. In the morning, the expression of *CO* is tightly repressed by CDFs physically binding to the DOF binding sites in the *CO* promoter [14, 15]. In early afternoon until dusk, F-box containing blue-light photoreceptor FLAVIN BINDING, KELCH REPEAT, F-BOX 1 (FKF1) forms a complex with GIGANTEA (GI) to degrade CDFs by the 26S proteasome-mediated protein degradation pathway [17]. Once the CDF repressor complex is removed from the *CO* promoter, FBHs bind to the *CO* promoter through E-box *cis*-elements, and strongly activate *CO* transcription [16]. The induction of *CO*, together with posttranslational stabilization of CO by multiple photoreceptors, induces *FT* expression towards dusk in long days [18–23]. However, the results from the mutant analysis of *fbh* and *cdf* quadruple mutants implied the existence of additional *CO* transcriptional regulator(s) especially in the afternoon, when the timing of *CO* expression is crucial for day-length discrimination [14, 16]. In order to more comprehensively understand how *CO* transcription is regulated, we extended our search for other regulators of *CO* transcription using a large-scale *Arabidopsis* transcription factor library [24]. We found additional transcription factors that can bind to the *CO* promoter, including the TEOSINTE BRANCHED 1/ CYCLOIDEA/ PROLIFERATING CELL NUCLEAR ANTIGEN FACTOR (TCP) transcription family.

The *TCP* genes are evolutionarily conserved plant-specific transcription factor genes, which are named after three of the originally identified members; *TEOSINTE BRANCHED 1* (*TB1*) in *Zea maize*, *CYCLOIDEA* (*CYC*) in *Antirrhinum majus*, and *PROLIFERATING CELL NUCLEAR ANTIGEN FACTOR 1* (*PCF1*) and *PCF2* in *Oryza sativa* [25–29]. The TCP protein possesses the non-canonical bHLH motif referred to as the TCP domain, which confers DNA binding and protein-protein interaction capabilities [25]. Based on the sequence variations in the TCP domains, *TCP* genes are largely classified into two subclasses: class I *TCP* and class II *TCP* [30–34]. The class II *TCP* genes are further categorized into two subgroups: the *CINCINNATA* (*CIN*)-like *TCP* (*CIN-TCP*) group ubiquitous in the plant kingdom, and the angiosperm-specific *CYC*/*TB1* group [29]. The *CIN-TCP* group consists of 8 members (*TCP2*, *3*, *4*, *5*, *10*, *13*, *17*, and *24*), and 5 of them are targeted by microRNA 319/JAW (miR319/JAW) [35]. CIN-TCPs function in a highly redundant manner to control lateral organ development, leaf senescence, and hormone signaling and biosynthesis [36–48]. In leaf development, CIN-TCPs have two functions. One is inhibiting cell proliferation in leaf marginal regions by regulating the cytokinin pathway and cell cycle regulatory genes, and the other is accelerating leaf aging by promoting the biosynthesis of jasmonic acid and upregulation of *WRKY53*, which positively regulates leaf aging [36, 46, 47, 49–51]. While loss of, or reduced function of *CIN-TCP*s caused the formation of serrated leaves and retained meristematic activity in the leaf margin, overexpression of the miRNA-resistant form of *TCP4* caused reduction in leaf size and earlier onset of leaf senescence [35, 40, 51]. Previously, it was reported that the *tcp4* mutation as well as reduced expression of related *CIN-TCP* genes caused late flowering phenotypes [36, 46]. In addition, overexpression of miRNA-resistant *TCP4* shortened the vegetative phase in adult plants and caused early flowering [51]. Although *CIN-TCP* genes are involved in flowering time regulation, the precise mechanism by which CIN-TCP proteins regulate flowering remained unknown.

Here we identified class II-type TCP4 and its related CIN-TCP transcription factors as new members of *CO* activators in *Arabidopsis*. TCP4 directly associated with the *CO* promoter through TCP binding sites and promoted *CO* expression around dusk, together with other *CO* activators, FBHs. In addition, we demonstrated that TCP4 physically interacted with GI, and activated *CO* in a *GI*-dependent manner. This genetic dependency of *TCP4* on *GI* to activate *CO* can be partially explained by the DNA binding ability of TCP4. Moreover, TCP4 interacted with all CIN-TCPs *in vivo*. Our results demonstrated that the interaction and functional dependency among *CO* regulators are important mechanisms for precise control of daily expression of *CO* transcription, which is an important regulation in the photoperiodic flowering pathway in *Arabidopsis*.

## Results

### The class II CIN-TCP family of proteins function as *CO* activators

**Fig 1:**
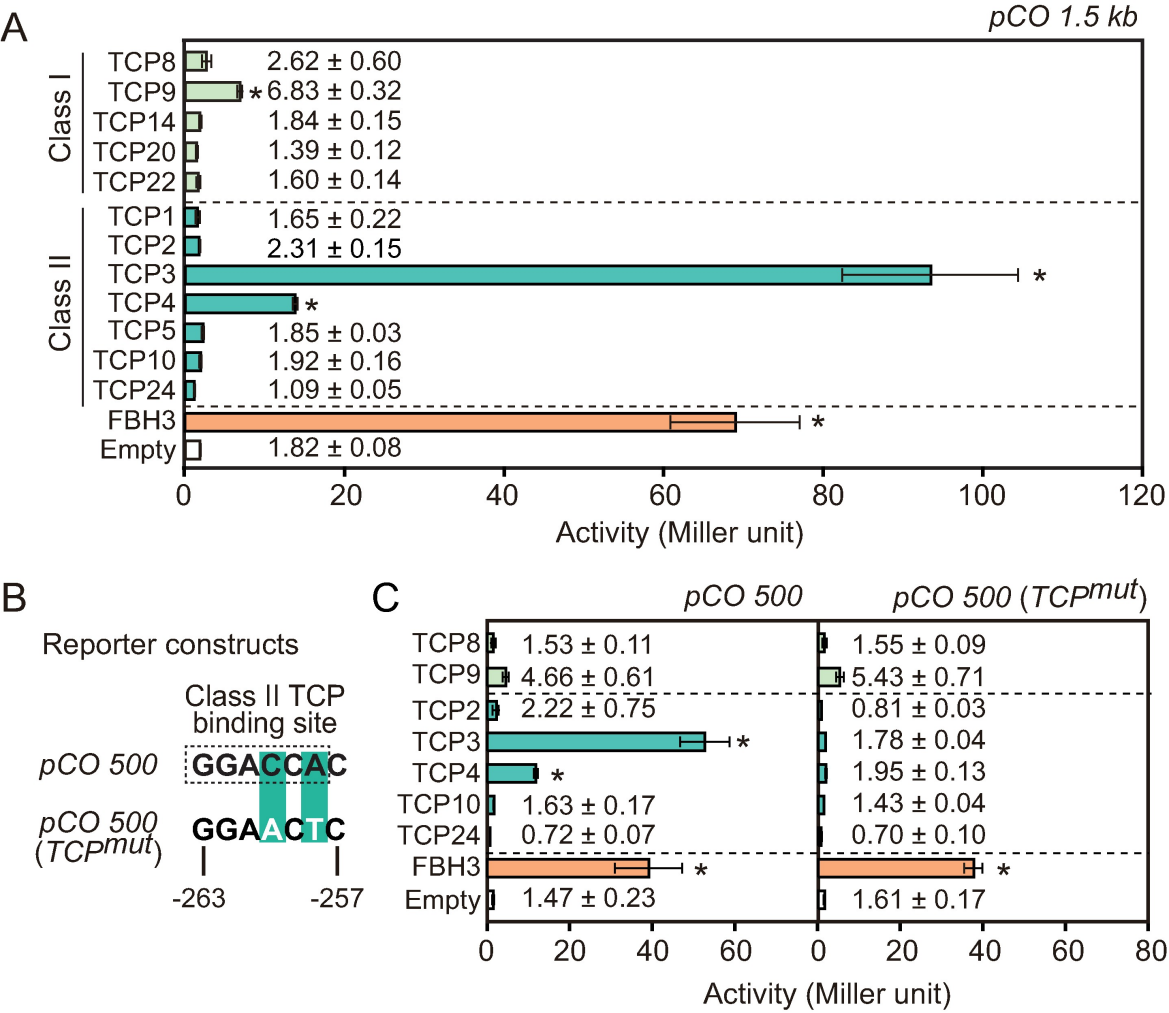
Class II TCP proteins bind to the *CO* promoter through the TCP binding site in yeast. (A) Binding of several members of both class I and class II TCP proteins to the *CO* promoter was analyzed in yeast. Bars and numbers represent β-galactosidase enzyme activity (Miller units) controlled by 1.5 kb of the *CO* promoter fragments. (B) A core sequence of the class II TCP binding site located within the 500-bp upstream region of the *CO* promoter and the TCP binding site mutation used in (C) are shown. The transcription start site is indicated as +1. (C) Interactions of class I and class II TCP proteins with the *CO* promoter with/without the TCP binding site were tested in yeast. Bars and numbers represent β-galactosidase enzyme activity (Miller units) controlled by 500 bp of the *CO* promoter fragment (*pCO500*, left) or the *CO* promoter with the TCP binding site mutated [*pCO500* (*TCP*^*mut*^), right]. Significant differences from the value of the control plasmid are indicated by asterisks (Bonferroni-corrected Welch’s *t*-test, *p*<0.05). Data represent means ± SEM (*n* = 4).

CO plays a critical role in the *Arabidopsis* photoperiodic pathway [2, 12, 13]. Although both transcriptional and posttranslational regulation of CO restricts its activity to long-day afternoons, the transcriptional regulation of *CO* has been less characterized. Currently, the *CDF* and *FBH* families of transcription factors are the only known regulators of *CO* [14–16]. To identify additional transcription factors that control *CO* transcription, we searched for ones that could induce the expression of *CO* promoter (1.5 kb)-driven *lacZ* reporter in yeast, using a comprehensive *Arabidopsis* transcription factor library [24]. Our screening identified 22 transcription factors (with more than two-fold induction than controls), including known *CO* regulators such as FBH3 and CDF2, as potential regulators of *CO* (S1 Table), validating the effectiveness of this approach. Other than bHLH transcription factors, bZIP transcription factors involved in ABA signaling [52–54] and TCP transcription factors [25] were over-represented in the candidate list (S1 Table). Since some *tcp* single and multiple mutants, including *tcp4*, showed later flowering phenotypes in LD [36, 46], and because TCPs are generally transcriptional activators [40, 41], we hypothesized that TCP might regulate flowering time in part by inducing *CO* transcription. In addition, the promoter sequence comparison of *CO* homologs in Brassicaceae revealed that the class II TCP binding site (GGACCA [30, 46]) was uniquely enriched among *CO* homologs and the positions of the elements were highly conserved [55]. This suggested the importance of the TCP contribution to the transcriptional regulation of *CO* at least in Brassicaceae. The *Arabidopsis* genome contains 24 *TCP* genes and these TCP proteins are functionally redundant within the same classes [31–34, 56]. Also, DNA binding sequences for class I and class II TCPs are distinct, but partially overlap [30]. We first confirmed the results of our large-scale yeast one-hybrid screening using some class I and II types of TCP clones in the yeast one-hybrid system (Fig 1A). High induction of *LacZ* expression regulated by *CO* promoter was induced in yeast by TCP3 and TCP4, both of which belong to the class II CIN-TCP clade [33], while moderate induction was observed with TCP9, which belongs to class I TCP. (Fig 1A). This induction was still observed when the *CO* promoter was shortened to the 500-bp fragment, in which one class II TCP binding site exists (Fig 1B and 1C). When the TCP binding site was mutated (*TCP*^*mut*^), the LacZ activity induced by the class II CIN-TCPs was completely abolished (Fig 1B and 1C), indicating that TCP3 and TCP4 directly bind to the *CO* promoter through the TCP binding site in yeast. On the other hand, induction of LacZ activity by TCP9 was not affected when the TCP binding site was mutated (Fig 1C). Because there is no canonical class I TCP binding site within this region of the *CO* promoter, we speculated that TCP9 may indirectly induce *LacZ* in yeast.

**Fig 2:**
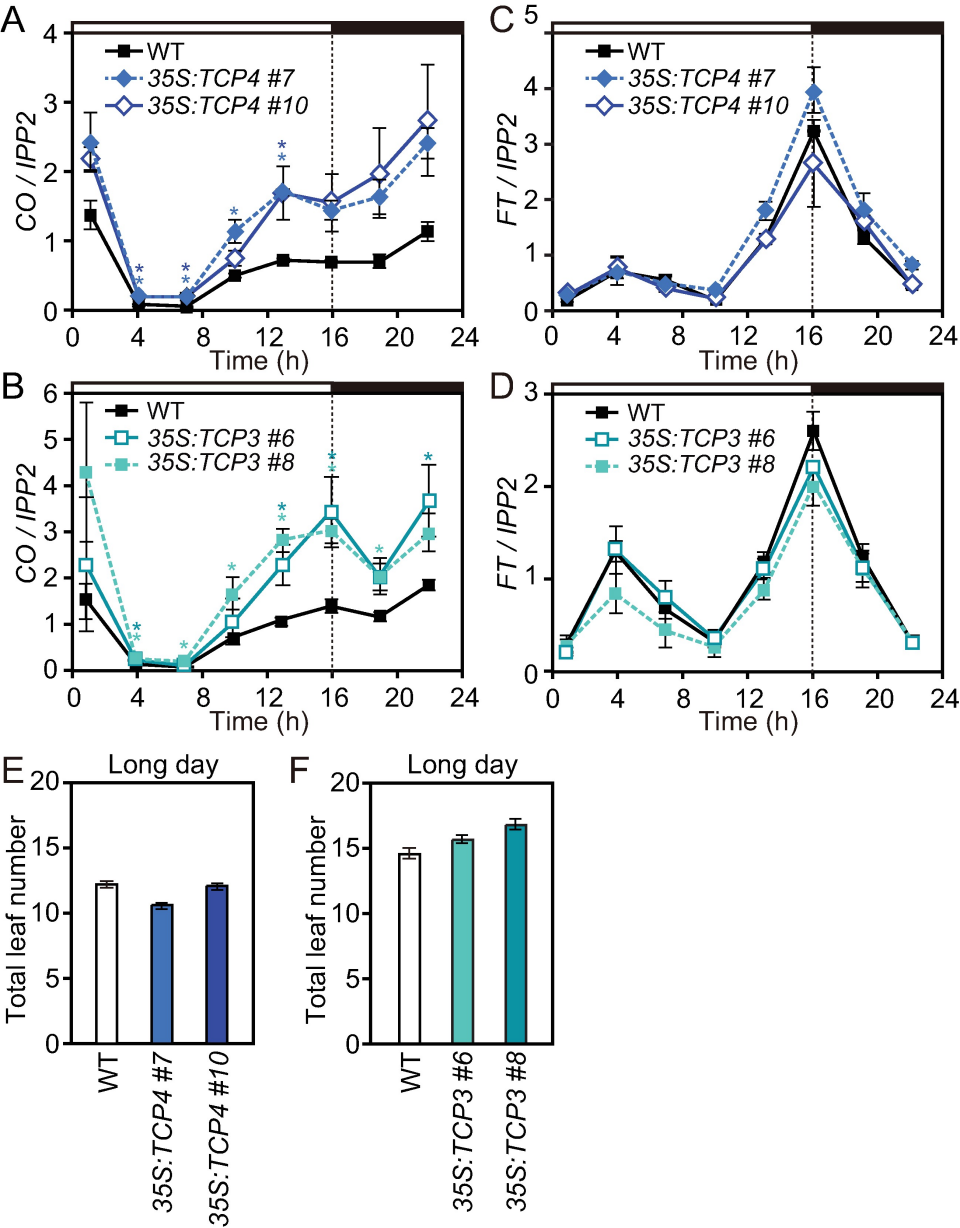
TCP4 and TCP3 activates the transcription of *CO*. (A to D) Gene expression patterns of *CO* (A and B) and *FT* (C and D) in LD in *35S*:*TCP4* (A and C), *35S*:*TCP3* (B and D) in comparison to wild type (WT) are shown. Bars above the traces represent light conditions; open and filled bars represent day and night, respectively. Time indicates hours (h) after light onset within a day. All expression data normalized against *IPP2* are shown relative to the average expression values of each gene in WT. Significant differences from WT are indicated by asterisks (*p<*0.05, Dunnett’s test). Data represent means ± SEM (*n* = 3). (E and F) Flowering phenotypes of the *35S*:*TCP4* (E) and *35S*:*TCP3* (F) plants in LD were analyzed. Total number of rosette leaves and cauline leaves generated from the main stem were counted when plants bolted. Data represent means ± SEM (*n*≥16).

We next investigated whether TCP3 and TCP4 function as *CO* activators *in vivo*. We generated transgenic plants in which either the *TCP3* or *TCP4* coding sequence was overexpressed (*35S*:*TCP3* and *35S*:*TCP4*, S1A to S1D Fig) to analyze the expression profiles of *CO* in LD and short days (SD). The *CO* expression levels in both the *35S*:*TCP3* and *35S*:*TCP4* lines were elevated several fold at its peak, without changing the overall diurnal expression patterns in LD and SD (Fig 2A and 2B, S1E and S1G Fig), indicating that both TCP3 and TCP4 can induce *CO* transcription. Even though we did not observe the clear binding of other class II CIN-TCPs to the *CO* promoter in yeast, overexpression of *TCP10*, which is a close homolog of *TCP3* and *TCP4*, also elevated the *CO* transcriptional levels (S1I and S1J Fig). These results imply that TCP3, TCP4, and TCP10 function as transcriptional activators of *CO*. We also analyzed the expression patterns of *FT* in these lines, and found that *FT* levels in these lines were similar to that in WT (Fig 2C and 2D, S1F and S1H Fig). CO protein is negatively regulated posttranslationally [22]. This result may indicate that the induction levels of *CO* mRNA in the *35S*:*TCP* lines were not sufficient to further increase the levels of CO, which consequently increased the *FT* levels. Consistent with the *FT* levels, flowering time of these lines resembled wild type plants (Fig 2E and 2F).

**Fig 3:**
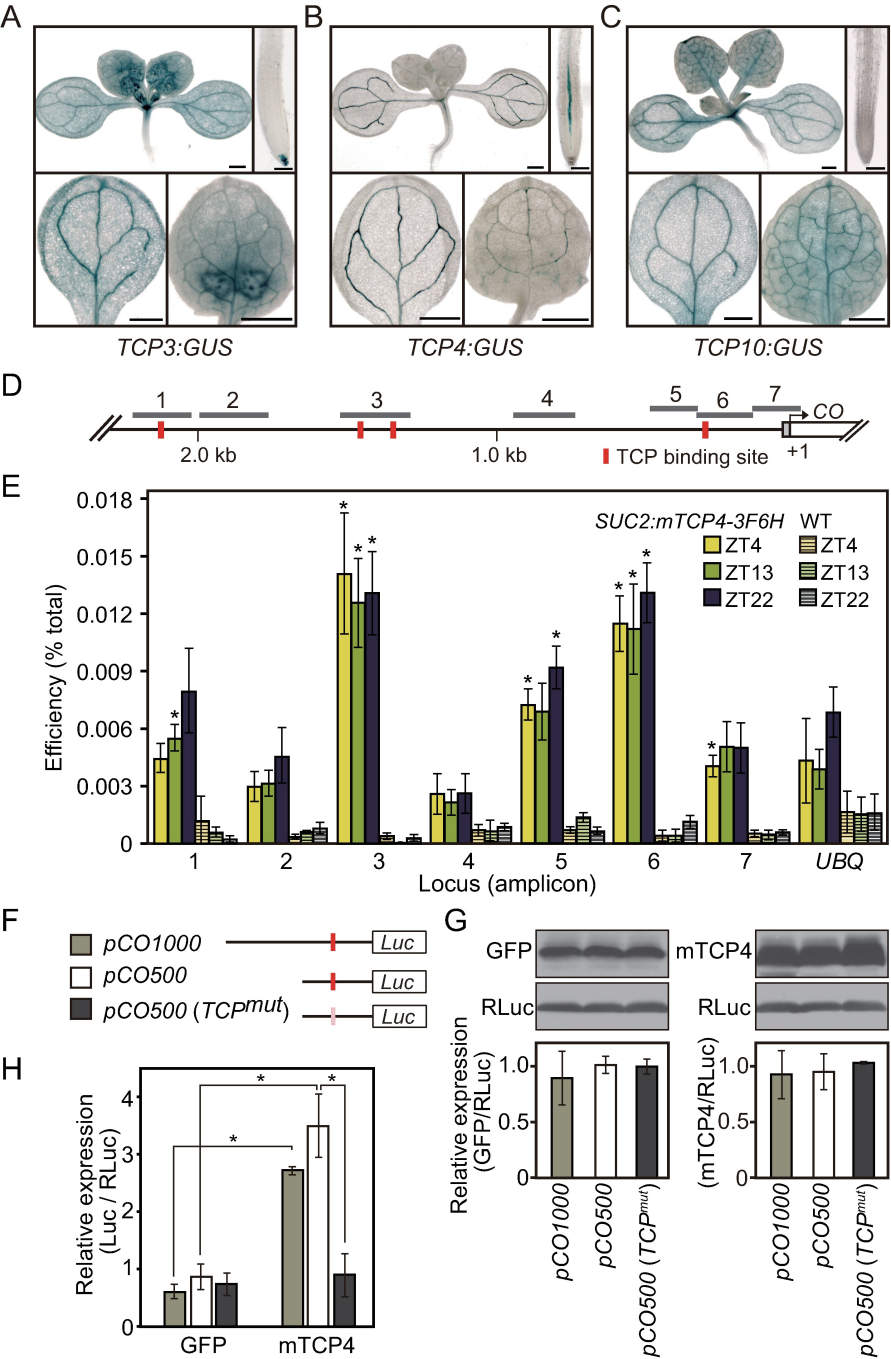
TCP4 physically associates with *CO* promoter and activates its transcription. (A to C) Histochemical GUS staining images from plants harboring the GUS reporter controlled by the class II *TCP* promoters. Images from whole seedlings, cotyledons, and the first set of leaves in *TCP3*:*GUS* (A), *TCP4*:*GUS* (B), and *TCP10*:*GUS* (C) plants grown in LD are shown. Scale bar, 0.5 mm. (D) A diagram of the *CO* locus and the locations of 7 amplicons used in ChIP analysis. The gray box represents 5′-UTR and the white box represents the first exon. Red boxes represent class II TCP binding sites (GGACCA). (E) Results of ChIP analysis using *SUC2*:*mTCP4-3F6H* plants against the *CO* promoter harvested at different times of day are shown. mTCP4 contains synonymous mutations in the miR319-binding site. 10-day-old plants grown in LD were harvested at Zeitgeber time (ZT) 4, 13, and 22. The *UBQ10* locus was used as a control. Significant differences from WT harvested on the same ZT are indicated by asterisks (one-tailed Student’s *t*-test, *p<*0.005). Data represent means ± SEM (*n* = 4). (F) A schematic diagram of different lengths of the *CO* promoter with the location of the TCP-binding site used in (H) is depicted. Red boxes represent TCP binding sites and a pink box represents a mutated TCP binding site (*TCP*^*mut*^). (G) The representative western blot images of effectors (GFP and mTCP4) and the reference [Renilla Luc (RLuc)] in each combination analyzed in (H) and quantitative results of the effector levels (relative to RLuc levels) obtained from 3 independent biological replicates are shown. (H) The results of the luciferase reporter assay in *N*. *benthamiana* are shown. The effects of *TCP4* on firefly luciferase (Luc) activities controlled by 1,000-bp of the *CO* promoter (gray bars), 500-bp (white bars), and 500-bp with a mutation on the TCP-binding site (dark gray bars) are tested. The activities of firefly Luc were normalized by the activities of RLuc. Asterisks denote significant differences in each combination (Bonferroni-corrected student’s *t*-test, *p<*0.05). Data represent means ± SEM (*n* = 3).

Our yeast one-hybrid assay results, as well as the increased *CO* levels observed in these *35S*:*TCP* lines, suggest that some of the class II TCPs (TCP3, TCP4, and TCP10, etc.) may function as *CO* activators *in vivo*. To assess this possibility, we studied whether these *TCP* genes are expressed in the same tissues where *CO* is expressed. We examined the spatial expression patterns of these *TCP* genes using histochemical GUS staining analysis. GUS activity in *TCP3*:*GUS*, *TCP4*:*GUS*, and *TCP10*:*GUS* plants were detected in leaf vascular tissues where *CO* is expressed (Fig 3A to 3C, [9, 40, 57]), indicating that these TCP proteins could exist where *CO* transcription occurs. Next, to investigate whether TCP binds to the *CO* promoter *in vivo*, we performed chromatin immunoprecipitation (ChIP) assay. We used TCP4 as a representative class II CIN-TCP, as it is the most characterized class II TCP protein [42, 46, 47, 51]. Because most class II *CIN-TCP* transcripts are regulated by miR319/JAW, we generated plants that express the miR319-resistant form of *TCP4* cDNA (*mTCP4* [35]) translationally fused to the 3xFLAG-6xHis (3F6H) epitope tag in phloem companion cells under the control of *SUCROSE-PROTON SYMPOTER 2* (*SUC2*) promoter (*SUC2*:*mTCP4-3F6H*, S2 Fig). Significant induction of *CO* was not observed in *SUC2*:*mTCP4-3F6H*, potentially due to a relatively low expression level of *mTCP4-3F6H* in this line (S2A and S2B Fig). ChIP-qPCR analysis showed that mTCP4-3F6H specifically associated with amplicons 1, 3, 5, 6, and 7 of the *CO* promoter, all of which contain or are adjacent to class II TCP binding sites (Fig 3D and 3E). TCP4 protein was constitutively expressed in the line (S2C Fig), and we did not observe clear changes in the binding of TCP4 to these regions among the time points analyzed (Fig 3D and 3E), indicating that there is no obvious time-dependent binding of TCP4 to the *CO* promoter.

To analyze whether the direct binding of TCP4 to the TCP binding site in the *CO* promoter is important for induction of *CO*, we utilized a *CO* promoter-controlled luciferase (Luc) reporter with/without the TCP binding site mutation in a transient expression system in *N*. *benthamiana* (Fig 3F). When a similar amount of TCP4 protein was expressed (Fig 3G), it significantly induced the expression of Luc reporter controlled by either about 1 kb or 500 bp of the *CO* promoters (*pCO 1000* and *pCO 500*), both of which contain one TCP binding site (Fig 3F and 3H). When the TCP binding site was mutated in the *pCO 500* reporter [*pCO 500* (*TCP*^*mut*^)], TCP4 was no longer able to induce the expression of the Luc reporter (Fig 3H). These results indicate that TCP4 activates the transcription of the *CO* promoter-controlled *Luc* reporter mainly through direct binding to the TCP binding site. Taken together, these results suggest that TCP4 associates with the *CO* promoter through the TCP binding sites to induce *CO* transcription *in vivo*.

**Fig 4:**
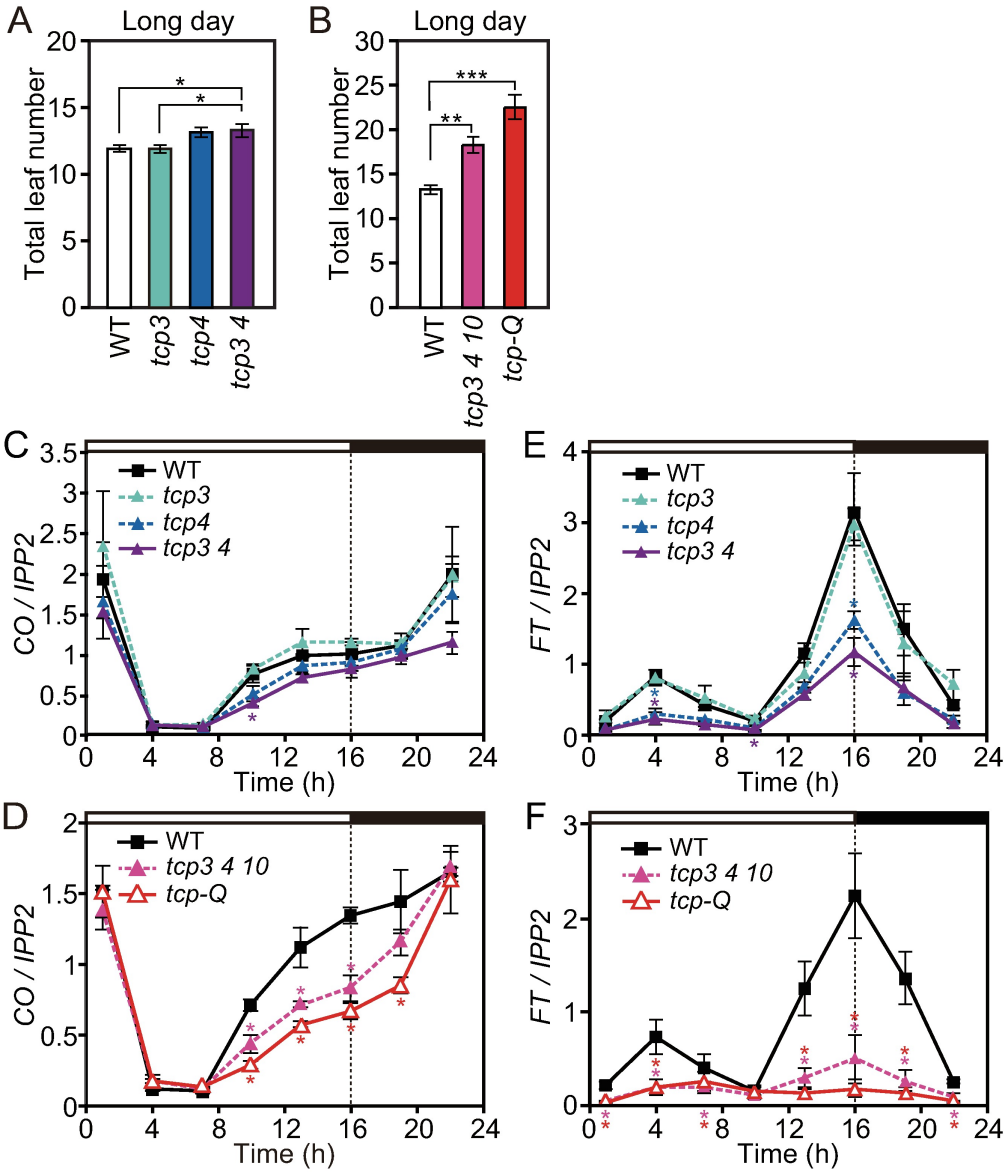
Class II CIN-TCP proteins redundantly function as transcriptional activators of *CO*. (A and B) Flowering phenotypes of single, double (A), and higher order *tcp* mutants (B) in LD were analyzed. Total number of rosette leaves and cauline leaves generated from the main stem were counted when plants bolted. Significant differences are indicated by asterisks (HSD test; *p<0.05, **p<0.01, ***p<0.001). Data represent means ± SEM (*n*≥16). (C to F) Gene expression patterns of *CO* (C and D) and *FT* (E and F) in LD in single, double (C and E), and higher order *tcp* mutants (D and F) are shown. Significant differences from WT values are indicated by asterisks (*p<*0.05, Dunnett’s test). Data represent means ± SEM (*n* = 3).

TCP4-related class II CIN-TCPs have overlapping functions in plant development [40, 56]. To confirm that class II TCPs, including TCP3 and TCP4, are involved in flowering regulation, we analyzed flowering phenotypes in single, double and higher order *tcp* mutants. In our growth conditions, the *tcp4* single mutant showed a quite subtle late-flowering phenotype in LD (Fig 4A). The degree of the late flowering phenotype became obvious in LD (but not in SD) when the mutations of related class II *TCP* genes were integrated into *tcp4* (Fig 4A and 4B and S3A Fig), indicating that the class II *TCP* genes redundantly regulate flowering time in LD. This is in agreement with the previous report showing that loss-of-function alleles for the *tcp4* single mutant or multiple class II CIN-TCP mutants caused late flowering in LD [36, 46]. We next analyzed the expression levels of *CO* and *FT* in these mutants. Although *CO* expression levels were not largely affected in single and double mutants of *tcp3* and *tcp4*, *CO* expression levels were significantly decreased in higher order *tcp* mutants [*tcp 3 4 10* and *tcp 3 4 5 10 13* (*tcp-Q*)] especially from afternoon to evening [Zeitgeber time 10 (ZT10) to ZT19] in both LD and SD (Figs 4C, 4D and S3B). These results suggest that TCP3, TCP4, and their closer homologs in the class II CIN-TCPs redundantly activate *CO* transcription from afternoon to evening. Peak *FT* expression level in the *tcp4* single mutant was reduced to almost two thirds of the wild-type level, but not in *tcp3* (Fig 4E). The *FT* levels were further reduced in the higher order *tcp* mutants in LD and in SD (Fig 4E and 4F and S3C Fig). These results indicate that class II TCP may work as an activator of *FT*. However, we did not find any class II TCP binding sites on the *FT* promoter, therefore, we predicted that the effects of class II *TCP* mutations on *FT* levels are likely indirect.

### TCP and FBH1 act additively to induce *CO*

**Fig 5:**
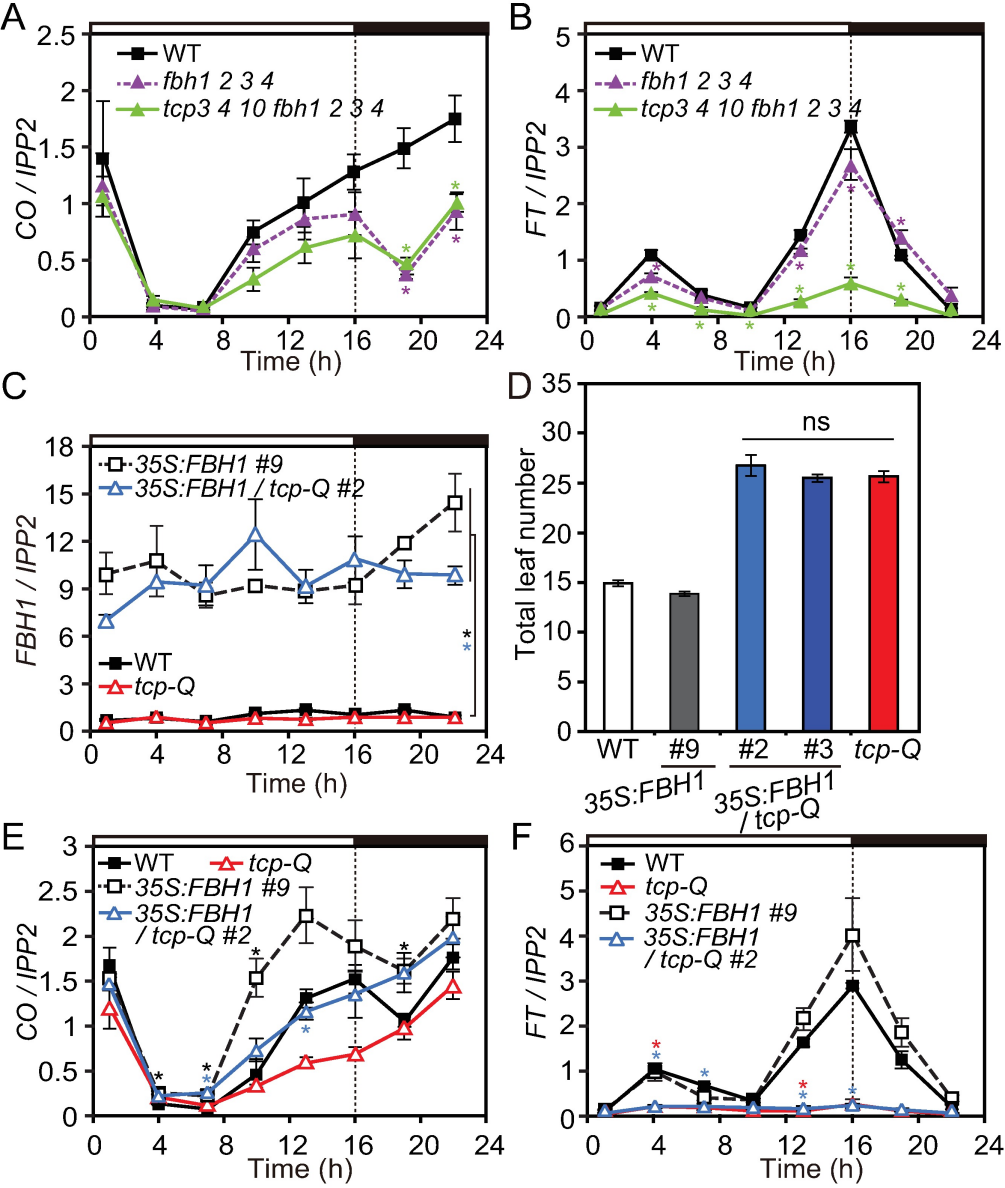
TCP and FBH coordinately regulate *CO* expression. (A to C, E and F) Gene expression patterns of *CO* (A and E), *FT* (B and F), and *FBH1* (C) in *fbh tcp* combinational mutants (A and B) and *35S*:*FBH1* with or without higher order *tcp* mutation (C, E and F) grown in LD are shown. For (A) and (B), significant differences from WT values are indicated by asterisks (*p<*0.05, Dunnet’s test). (D) The flowering phenotype of the *35S*:*FBH1* plants grown in LD. Total number of rosette leaves and cauline leaves generated from the main stem were counted when plants bolted. ns: no significance (HSD test). Data represent means ± SEM (*n*≥15). For (C), (E) and (F), significant differences between the *35S*:*FBH1* lines and their background strains are indicated by asterisks (*p<*0.05, Student’s *t*-test). Data represent means ± SEM (*n* = 3).

Our results imply that some class II CIN-TCPs (such as TCP3, TCP4, and TCP10) are transcriptional activators of *CO*. As we previously identified the other group of *CO* transcriptional activators, FBHs [16], we next aimed to analyze the functional relationship between two groups of *CO* transcriptional activators, TCPs and FBHs. We first generated the *tcp fbh* septuple mutant in which three similar *TCP*s (*tcp 3*, *4*, and *10*) and four *FBH*s (*fbh1*, *2*, *3*, and *4*) were mutated, and analyzed *CO* expression patterns in the mutant in LD (Fig 5A and S4 Fig). The *CO* expression levels during the afternoon in the *tcp fbh* septuple mutant were slightly lower than those in the *fbh* quadruple mutant (Fig 5A). This suggests that *FBH* and *TCP* regulate *CO* in an additive manner during LD afternoon, whereas *FBH*s, but not *TCP*s, play major roles in *CO* transcription at night. We also analyzed *FT* expression in these lines. While the *FT* levels were not much affected in the *fbh* quadruple mutant, introducing three *tcp* mutations largely repressed *FT* in the *tcp fbh* mutant (Fig 5B), which is similar to the phenotype of the *tcp 3 4 10* triple mutant (Fig 4F). To further investigate the genetic relationship between *FBH1* and *CIN-TCP*s, we generated lines that overexpressed similar levels of *FBH1* in WT and *tcp-Q* background (Fig 5C and S5A Fig). Overexpression of *FBH1* in *tcp-Q* completely failed to accelerate flowering time, and the *35S*:*FBH1/tcp-Q* plants showed a similar flowering phenotype to the *tcp-Q* plants (Fig 5D). To investigate the molecular mechanism underlying this phenotype, the expressions of *CO* and *FT* were analyzed. *FBH1* was still able to activate *CO* in the *tcp-Q* background, although the induction was weakened in the afternoon (Fig 5E and S5B Fig). Together with the additive effect of *fbh* and *tcp* mutations on the *CO* expression profile during daytime in LD (Fig 5A), these results suggest that *CIN*-*TCP*s and *FBH*s additively induce *CO* transcription mainly during the afternoon in LD. In contrast, changes in *FBH1* expression in the *tcp-Q* background had little effect on *FT* expression (Fig 5F and S5C Fig). Similar to the minimal change in *FT* expression observed in *35S*:*TCP* lines (Fig 2C and 2D), the induction of *CO* levels in the *35S*:*FBH1* lines might not be high enough to further induce *FT*. In addition, the epistatic effect of higher order *tcp* on *FT* expression in *fbh tcp* septuple and in the *35S*:*FBH1/tcp-Q* lines suggest that *CIN-TCP*s may have unknown indirect roles in the induction of *FT*, which may be independent of *CO* transcriptional regulation.

### TCP4 activates *CO* in *GI*-dependent manner

**Fig 6:**
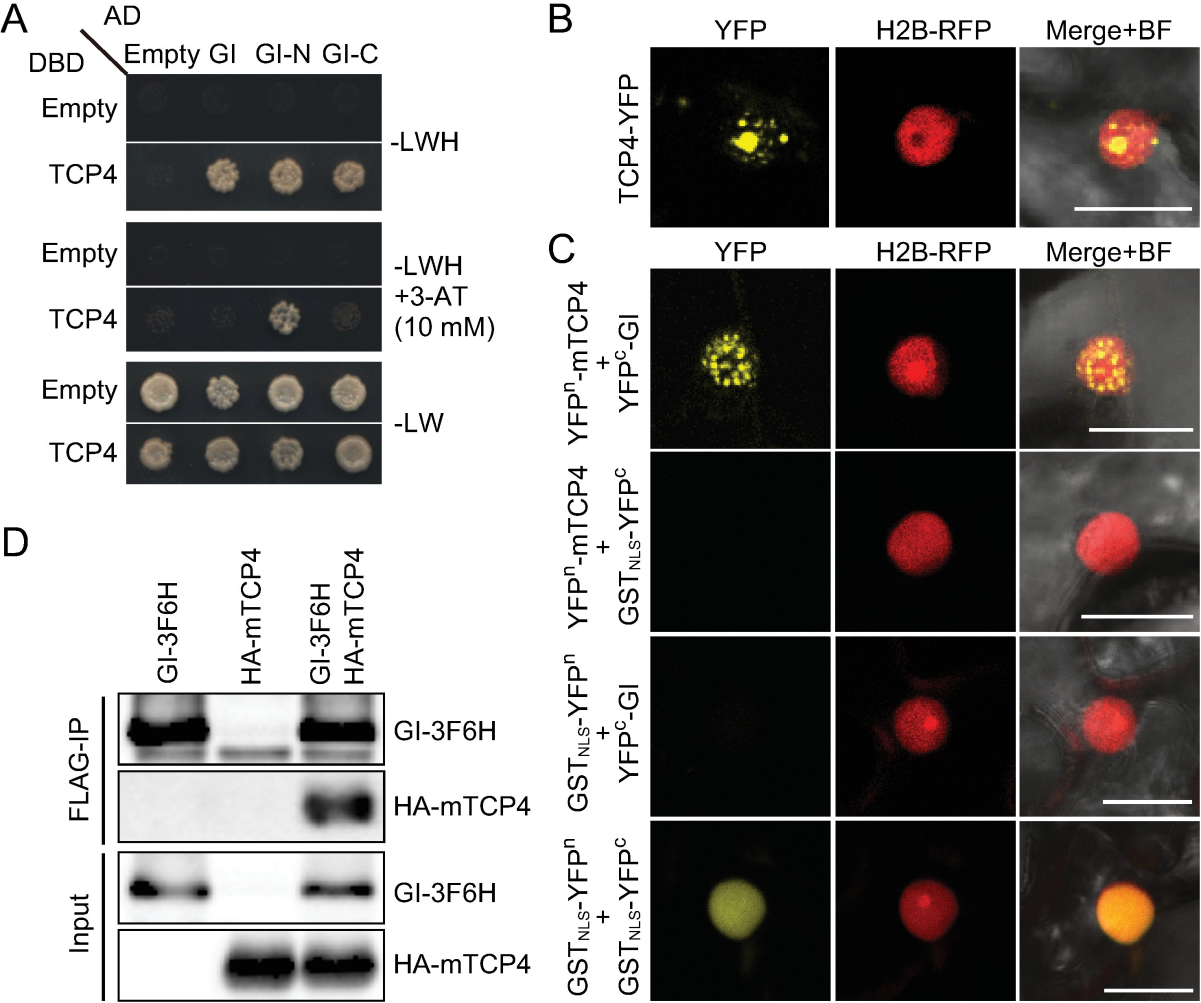
TCP4 physically interacts with GI. (A) Protein-protein interaction between TCP4 and GI was analyzed in yeast. Full-length TCP4 and full-length or truncated GI fused to the DNA-binding domain (DBD) or the activation domain (AD) of Gal4 were tested under selective [–LWH (top),–LWH+10 mM 3-aminotriazole (3-AT, middle)] and non-selective [–LW (bottom)] conditions. For GI, N and C indicate the amino acid residues 1–391 and 382–1173, respectively. (B) Subcellular localization of TCP4 fused to enhanced YFP (TCP4-YFP) was observed in *N*. *benthamiana* epidermal cells. H2B-RFP was used for the nuclear marker. Images from YFP and RFP channels were merged with bright-field (BF) images. (C) BiFC assays of interaction between mTCP4 and GI are shown. The full-length of mTCP4 fused to the N-terminal half of enhanced YFP (YFP^n^-mTCP4) and the full-length of GI fused to the C-terminal half of enhanced YFP (YFP^c^-GI) were co-expressed in *N*. *benthamiana* leaf epidermal cells. Nuclear-localized form of GST fragment (GST_NLS_) fused to YFP^n^ or YFP^c^ was used as negative control. Scale bar, 20 μm. (D) Co-immunoprecipitation (Co-IP) assay of HA-mTCP4 and GI-3F6H was performed. Proteins were expressed in *N*. *benthamiana*.

To investigate whether other known factors that regulate *CO* transcription also work together with TCP4 and/or FBH1, we performed yeast two-hybrid analysis and found that GI interacts with both TCP4 and FBH1 mainly through its N-terminal region (Fig 6A and S6A Fig). To validate these interactions in plant cells, we performed a bimolecular fluorescence complementation (BiFC) assay in *N*. *benthamiana* leaves. When TCP4-YFP was expressed *in planta*, fluorescent signals of TCP4-YFP localized in nuclear speckles and nucleoli (Fig 6B). Reconstituted split YFP signals derived from the combination of YFP^n^-mTCP4 and YFP^c^-GI constructs were also observed in nuclear speckles (Fig 6C). A broad nuclear signal (without nucleoli) was observed when GI-YFP^n^ and FBH1-YFP^c^ were co-infiltrated (S6B Fig). None of these constructs reconstituted YFP signals when co-infiltrated with the nuclear-localized form of GST (GST_NLS_) fused to YFP^n^/YFP^c^ (Fig 6C and S6B Fig). As a control, YFP^n^/YFP^c^ fused GST_NLS_ proteins dimerized in the nucleus (Fig 6C), as GST forms a dimer [58]. These results suggest that GI physically interacts with TCP4 and FBH1 in the nucleus. Physical interaction between TCP4 and GI was also confirmed by co-immunoprecipitation assay *in planta* (Fig 6D).

**Fig 7:**
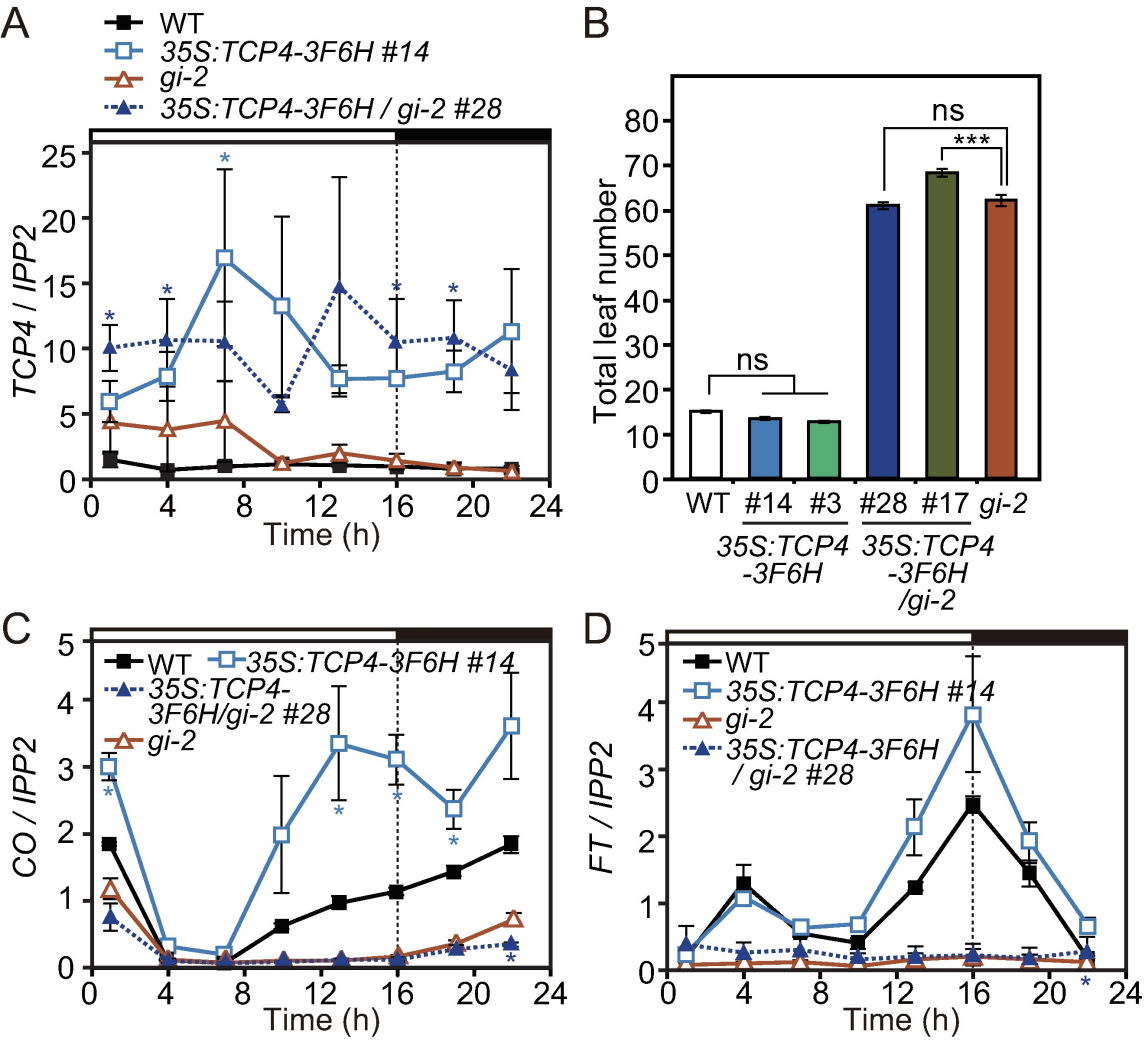
TCP4 activates *CO* in a *GI-*dependent manner. (A, C and D) Gene expression patterns of *TCP4* (A), *CO* (C), and *FT* (D) in WT, *35S*:*TCP4-3F6H*, *35S*:*TCP4-3F6H/gi-2*, and *gi-2* grown in LD are shown. Significant differences in values between the *35S*:*TCP4-3F6H* lines and their background strains are indicated by asterisks (*p<*0.05, Student’s *t*-test). Data represent means ± SEM (*n* = 3). (B) Flowering phenotype of the *35S*:*TCP4-3F6H* plants in LD. Significant differences are indicated by asterisks (HSD test; ns, no significance, *** *p<*0.001). Data represent means ± SEM (*n*≥14).

To assess the contribution of GI to FBH1 and TCP4 functions, we generated transgenic lines in which similar amounts of *FBH1* or *TCP4* are expressed with or without the *gi* mutation (Fig 7A, S6C and S7A Figs). As previously reported, *35S*:*FBH1* flowered slightly early in LD (S6D Fig [16]). The *35S*:*FBH1/gi-2* plants flowered earlier than the *gi-2* mutant but later than the *35S*:*FBH1* plants (S6D Fig). In *35S*:*FBH1/gi-2*, *CO* induction was largely compromised as compared to that in *35S*:*FBH1* (S6E and S6F Fig). However, especially at night (from ZT16 to ZT1), *FBH1* was still able to partially activate *CO* in the *gi-2* background (S6E and S6F Fig). *FT* expression in *35S*:*FBH1*/*gi-2* was almost identical to that in *gi-2* (S6G Fig). As *35S*:*FBH1*/*gi-2* flowered earlier than *gi-2*, we wondered whether other floral integrator genes may be expressed higher in *35S*:*FBH1*/*gi-2* than in *gi-2*. We therefore analyzed the expression levels of *SUPPRESSOR OF OVEREXPRESSION OF CONSTANS 1* (*SOC1*), which is one of the major floral integrators downstream in the photoperiodic pathway [8, 59–61]. In *35S*:*FBH1/gi-2*, the expression of *SOC1* was significantly upregulated compared to that in *gi-2* (S6H Fig). This may contribute to accelerated flowering time in the *35S*:*FBH1/gi-2* plants compared to the *gi-2* mutant. These results suggest that *GI* is not essential for *FBH1* to activate *CO*, although *GI* affects FBH1 activity.

Next, we analyzed the effect of *TCP4* overexpression in the *gi-2* background. In contrast to the phenotype observed in *35S*:*FBH1/gi-2*, the flowering phenotype of the lines expressing *35S*:*TCP4-3F6H* in the *gi-2* background were almost identical to *gi-2* (Fig 7B). In addition, *CO* induction observed in *35S*:*TCP4-3F6H* was completely abolished in *35S*:*TCP4-3F6H/gi-2* (Fig 7C and S7B Fig). *FT* expression profiles in the *35S*:*TCP4-3F6H/gi-2* lines were identical to those in the *gi-2* mutant (Fig 7D and S7C Fig). These results suggest that *TCP4* requires *GI* to activate *CO*. Taken together, these results indicate that TCP4 physically interacts with GI to activate *CO* in a *GI*-dependent manner, whereas *FBH1* is partially independent of *GI* to activate *CO*.

### GI enhances TCP4 binding onto the *CO* promoter

**Fig 8:**
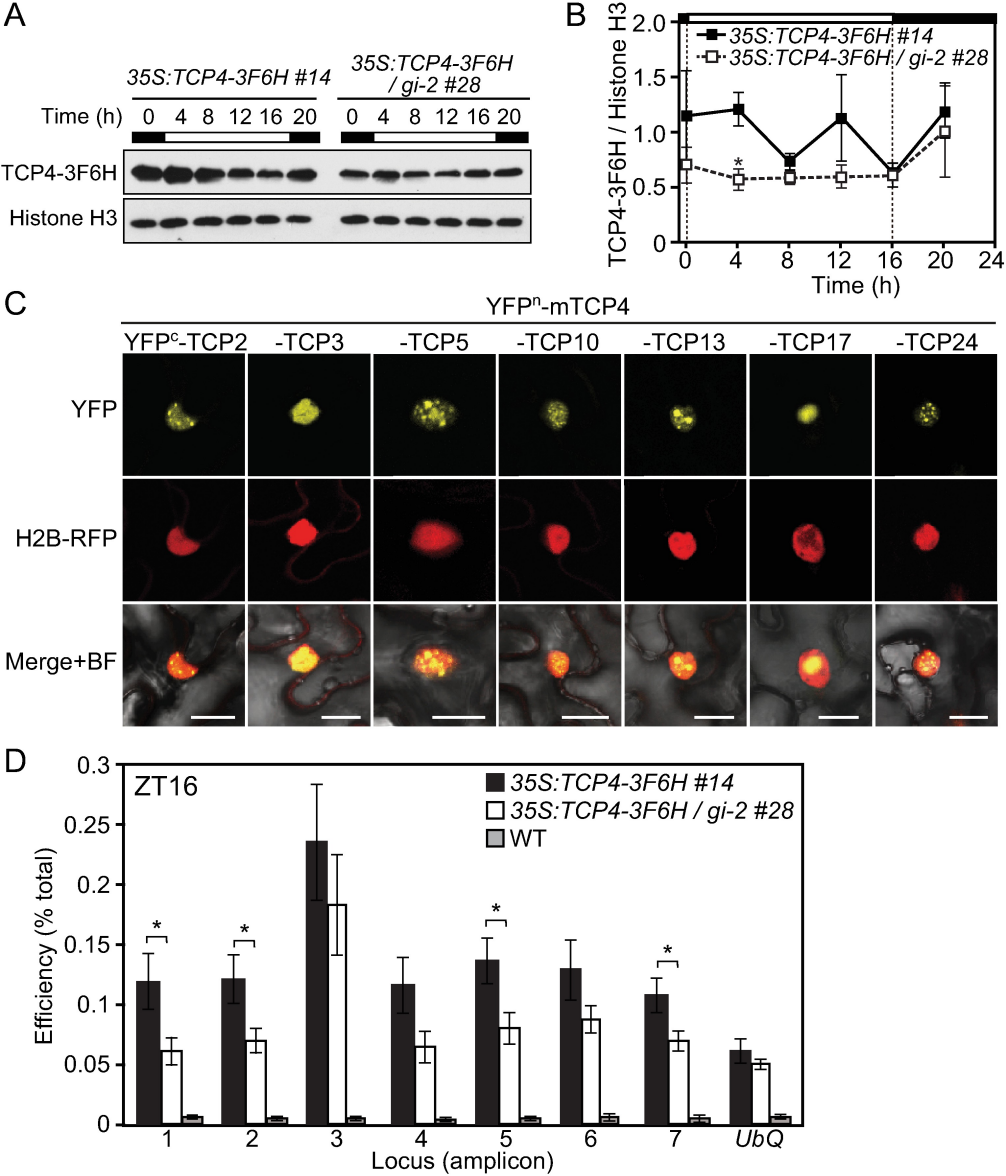
GI enhances TCP4-binding to the *CO* promoter. (A) The representative protein profiles of TCP4-3F6H overexpression in WT and *gi-2* background grown in LD are shown. Histone H3 protein was used as the loading control. The numbers above the images indicate time (h) after light onset within a day. (B) The quantified results of the TCP4-3F6H protein profiles shown in (A) are obtained from 3 independent biological replicates. Significant differences are indicated by asterisks (Student’s *t*-test, *p<*0.05). Data represent means ± SEM. (C) BiFC assay results of interactions among CIN-TCP proteins are shown. The full length of TCP2, 3, 5, 10, 13, 17, and 24 fused to YFP^c^ were co-expressed with YFP^n^-mTCP4 in *N*. *benthamiana* leaf epidermal cells. Scale bar, 20 μm. (D) Results of ChIP analysis using *35S*:*TCP4-3F6H* in either WT and *gi-2* backgrounds harvested at ZT16 are shown. Amplicons located in the *CO* promoter are described in Fig 3D. Significant differences are indicated by asterisks (Student’s *t*-test, *p<*0.05). Data represent means ± SEM (*n* = 4).

As our genetic analysis indicates that TCP4-dependent induction of *CO* may require functional GI, we sought to elucidate the mechanism by which GI directly regulates the function of TCP4 for *CO* transcriptional regulation. GI functions as a molecular hub to connect various environmental signals into the flowering pathway [62–65]. GI often affects the stabilities of its interacting proteins [14, 17, 66, 67]. In addition, a recent report showed that GI has a general chaperone activity and facilitates proper protein folding in its interacting partners [68]. Moreover, GI interacts with several transcription factors, which function in the flowering pathway, in the nucleus and also physically associates with the *CO* and *FT* promoter regions [17, 69]. Therefore, we speculated the following three possibilities to explain how GI regulates TCP4 activity. 1) GI may stabilize TCP4 protein. 2) GI may recruit other components that are important for the transcriptional activity of TCP4. 3) GI may enhance the DNA-binding of TCP4 to the *CO* promoter. To test the first possibility, we analyzed the daily expression profiles of TCP4 protein in the *35S*:*TCP4-3F6H* lines with/without the *gi* mutation. Although TCP4-3F6H protein tended to accumulate slightly less in the *gi-2* mutant background than in WT background, the overall protein amount was not significantly different between the two genetic backgrounds (Fig 8A and 8B, S7D and S7E Fig).

To test the second possibility, we next investigated whether GI affects the composition of the TCP4 complex. We performed tandem affinity purification coupled with mass spectrometry (TAP-MS) analysis to identify components of TCP4 complex *in vivo*, and explored the effect of the loss of *gi* on the composition. We purified a functional TCP4-3F6H protein complex from 2-week-old sample (*35S*:*TCP4-3F6H* and *35S*:*TCP4-3F6H* /*gi-2*) lines harvested at ZT13 when the daytime *CO* peaks. We identified peptides derived from approximately 30 different proteins as TCP4 interacting proteins (S2 Table). Among those, all the transcription factors identified belonged to the class II CIN-TCP family (Table 1). We confirmed these interactions by BiFC assay, showing that TCP4 interacted with all class II CIN-TCP proteins in the nucleus (Fig 8C and S8 Fig). Most combinations between mTCP4 and class II CIN-TCPs (except for mTCP4-TCP3 and occasionally mTCP4-TCP17 interactions) formed nuclear speckles (Fig 8C). Previously, mainly based on co-expression and yeast two-hybrid analyses, it was suggested that TCP may work as heterodimers formed among proteins belonging to the same class [56]. Our TAP-MS and BiFC results nicely validated the presence of multiple heterodimers *in vivo*. Our TCP4 interactor list did not contain GI, indicating that GI may not be a major interactor of TCP4. Also, we did not find a significant difference in the composition of the TCP4 complex between the WT and *gi-2* backgrounds (Table 1 and S2 Table), suggesting that interactions between TCP4 and other CIN-TCPs were not affected by the presence of GI. The MS analysis also provided information regarding the presence of protein modifications such as phosphorylation and ubiquitination. We identified nine phosphorylated residues and two ubiquitinated residues in the TCP4 peptides (S9 Fig and S3 Table). Interestingly, one of the clusters of phosphorylation sites matches the consensus of the casein kinase II phosphorylation sites (S/T-X-X-D/E), indicating that TCP4 may be phosphorylated by casein kinase II *in vivo*. We further analyzed whether GI modulates these post-translational modifications of TCP4. None of these modifications showed a clear difference between WT and *gi-2* background (S3 Table). These results indicate that, potentially, changing protein complex composition or protein modification of TCP4 is not how GI regulates TCP4 activity.

**Table 1:** TCP4 and its interacting class II CIN-TCP proteins identified by TAP-MS analysis.

		Estimated molecular mass (kDa)	No. of peptides*		
			*35S*:*TCP4-3F6H*^†^		WT
AGI no.	Name		WT#14	*gi-2* #28	
AT3G15030	TCP4	45.96	747	642	7
AT2G31070	TCP10	40.38	184	179	3
AT1G30210	TCP24	36.38	97	96	0
AT4G18390	TCP2	40.18	62	78	0
AT1G53230	TCP3	43.04	49	59	0
AT3G02150	TCP13	39.61	38	37	0
AT5G60970	TCP5	40.16	34	39	0
AT5G08070	TCP17	27.41	3	5	0

*Total number of peptides identified in 3 biological replicates of MS analysis (See details for S2 Table). WT sample was used as the negative control.

^†^No significant difference between WT and *gi-2* was detected in the number of peptides obtained.

To assess the third possibility, we tested whether GI affects the DNA binding of TCP4 by performing ChIP assay at ZT16, when the amount of TCP4-3F6H protein in WT and *gi-2* background was similar (Fig 8A and 8B). We found that the binding of the TCP4-3F6H protein to the *CO* promoter was reduced in *gi-2* on amplicons 1, 2, 5, and 7 (Fig 8D). Less binding of TCP4-3F6H to the *CO* promoter in *gi-2* was also observed on amplicon 1 at ZT13 (S10 Fig). Notably, both at the ZT13 and ZT16 time points, significant differences in the DNA binding were observed in the distal part of the TCP binding site (amplicon 1), where the highest amount of GI bound within the *CO* promoter [17]. These results suggest that GI may bind to TCP4 on the *CO* promoter to enhance DNA-binding of TCP4, which may play an important role in the activation of *CO* transcription. Although GI may enhance TCP4 binding to specific regions in the *CO* promoter, DNA binding of TCP4-3F6H was not completely lost in *gi-2*. This also suggests that there may be other unknown mechanisms of GI that contribute to the epistasis effect of the *gi-2* mutation on TCP4-dependent *CO* transcriptional regulation.

## Discussion

### TCP4 and its close homologs function as transcriptional activators of *CO*

Transcriptional regulation of *CO* is one of the key steps in the photoperiodic flowering pathway [3, 11]. Comparative analysis of the promoter regions of the *CO/COL* genes in Brassicaceae revealed that the *CO* promoter contains three blocks of highly conserved regions, and these regions are involved in generating daily expression patterns of *CO* and flowering time regulation [55]. Within these regions, several *cis*-elements were particularly conserved. These were Dof binding sites, class II TCP binding sites, and E-box elements [55]. It was previously shown that CDFs and FBHs bind to Dof binding sites and E-box elements, respectively [14–16]; however, it remained elusive whether and how class II TCPs regulate *CO* transcription. In addition, misregulation of *TCP4* affected flowering time, but the underlying mechanism was unknown [36, 46, 51]. In this study, we demonstrated that TCP4 and other class II CIN-TCPs are novel transcriptional activators of *CO* in *Arabidopsis*. TCP4, and likely other class II CIN-TCPs, physically associate with the *CO* promoter through multiple TCP binding sites, and promote *CO* expression mainly around dusk (Figs 1 to 4 and S1 Fig). Previously, based on protein-protein interaction in heterologous systems, structural analysis, and co-expression analysis, it was proposed that TCP4 forms a stable homodimer as well as heterodimers with other CIN-TCPs [30, 56, 70]. We successfully demonstrated that functional TCP4 forms heterodimers with all members of class II CIN-TCPs *in vivo* using a TAP-MS approach and a BiFC assay (Fig 8 and Table 1). Some potential TCP4-interacting proteins are chloroplast-localized proteins (S2 Table). A previous report showed that TCP13 is localized in chloroplasts and regulates expression of genes encoded in the chloroplast genome [71]. Although we did not observe either a TCP4-YFP signal or TCP4-TCP13 direct interaction in chloroplasts (Figs 6 and 8), potentially, a small portion of TCP4 may also be localized and function in chloroplasts, if these interactions are reproducible. Structural analysis of the TCP domain of TCP4 showed that mutations in the domain, which are located in a predicted dimer interface, abolish the DNA binding ability of TCP4 and fail to rescue *tcp4* mutant phenotype, indicating that dimerization plays an important role in its function [70]. We also found that miR319-targeted homologs of CIN-TCPs are present more abundantly in the TCP4 complex (Table 1). TCP10, which was the most abundant class II CIN-TCP found in the TCP4 complex, is predicted to function in a highly redundant manner with TCP4 (Table 1) [56]. BiFC analysis indicated that TCP4 interacts with class II CIN-TCPs and GI in nuclear speckles (Figs 6 and 8). These results indicate that TCP4 preferentially interacts with functionally redundant homologs of class II CIN-TCPs in the specific foci within the nucleus to regulate *CO* transcription as well as leaf development [35, 39–41, 44, 56].

### Functional network among *CO* regulators

In this study, we sought to investigate the regulatory network of *CO* regulators, especially among the positive regulators, TCP4, FBH1, and GI. Our results derived from genetic analysis using higher orders of *tcp fbh* mutants demonstrated that TCPs and FBHs additively activate *CO* transcription during the daytime, especially from ZT4 to ZT16 time points (Fig 5 and S5 Fig). BiFC analysis suggested that both FBH1 and TCP4 interact with GI in nucleus, but interaction patterns were not completely identical (Fig 6 and S6 Fig). In addition, FBH1 and TCP4 showed a difference in functional dependency on *GI*. While the presence of functional GI is not essential for FBH1-dependent induction of *CO*, TCP4 seemed to require GI function to activate *CO* transcription (Fig 7, S6 and S7 Figs). Therefore, FBH1 and TCP4 may regulate *CO* through different mechanisms although both of them interact and work together with GI.

Interestingly, overexpression of neither FBHs nor TCPs drastically changed the temporal expression patterns of *CO*, although the amplitude of *CO* expression was increased (Figs 2, 5 and 7 and S5 to S7 Figs) [16]. In contrast, overexpression of *CO* repressors, CDFs, and loss-of function of *cdf* multiple mutants can change the daily expression patterns of *CO* [14, 15]. This suggests that repressors may play a more important role in creating the daily expression patterns of *CO*, while activators ensure that a certain amount of *CO* is transcribed near dusk. A similar mechanism is proposed in the plant circadian clock, where activators are not essential for generating circadian oscillation by themselves, but they can confer the robustness of the oscillation under a wide range of environmental conditions [72, 73].

### TCP4 requires GI to activate *CO* transcription

Our results demonstrated that TCP4 physically interacts with GI and activates *CO* in a *GI*-dependent manner (Figs 6 and 7 and S7 Fig). In addition, TCP4 and GI partially share their target sites on the *CO* promoter (amplicons 1, 5, and 6) [17] (Figs 3 and 8 and S10 Fig). Furthermore, the amount of TCP4 associated with the specific regions of the *CO* promoter was reduced in *gi-2*, especially where GI was most abundant on the *CO* promoter (Fig 8 and S10 Fig) [17]. We propose that TCP4-GI complex formation may be recruited to the class II TCP binding sites on the *CO* promoter and/or stabilize the association of TCP4 with the *CO* promoter. Recently, it was shown that GI acts as a co-chaperone with HSP90 to facilitate the maturation of its interacting partner, ZEITLUPE (ZTL) [68]. It might be possible that the *gi-2* mutation partially affects TCP4 protein maturation to maintain its association with the *CO* promoter or to fully function as a *CO* transcriptional activator. ZTL protein abundance is significantly reduced in the *gi* mutants likely due to the destabilization of misfolded ZTL protein [68]. However, the protein levels of TCP4 were not largely affected by the *gi-2* mutation (Fig 8 and S7 Fig), indicating that, even though GI may potentially affect TCP4 protein maturation, the effect of GI on TCP4 protein is different from that on ZTL. In LD, TCP4 induced *CO* expression mainly from mid-day to dusk (Figs 2 and 4). Although the expression of *TCP3* and *TCP4* peaks around ZT10 to 13 in LD (S1 and S2 Figs), both protein stability and DNA binding ability of TCP4 are almost consistent at ZT4, ZT13, and ZT22 (Fig 3 and S2 Fig). On the other hand, transcriptional and post-translational regulation of *GI* results in GI protein accumulation towards dusk [17, 74, 75]. In addition, GI changes subnuclear localization throughout the day [76]. These potential time-dependent functions of GI may enhance the activity of TCP4 at specific times of day.

Other than homo- and hetero-dimerization of TCPs, TCPs interact with other proteins as well. In the cytokinin signaling pathway, TCP4 requires chromatin remodeling factor BRAHMA to activate the expression of *ARR6* [49]. In addition, CIN-TCPs interact with the transcriptional repressor TCP INTERACTOR CONTAINING EAR MOTIF PROTEIN 1 (TIE1), which also interacts with co-repressor TOPLESS, and this interaction modifies the transcriptional activities of CIN-TCPs [77]. Another member of CIN-TCP (TCP24) interacts with ARMADILLO BTB ARABIDOPSIS PROTEIN1, which may function as a member of ubiquitin E3 ligase [78, 79]. Our TAP-MS analysis of the *in vivo* TCP4 complex identified members of class II CIN-TCPs as TCP4 interactors, but neither chromatin remodeling factors nor other components involved in transcriptional machineries were identified (S2 Table). Similarly, GI was not in the list of the interactors (S2 Table). The TAP procedure is composed of dual affinity purification processes to reduce false positives; therefore, it is designed to detect strong and major protein-protein interactions [80, 81]. This implies that interactions with those potential known co-regulators occur under very limited conditions or transiently, or that the expression levels of the co-regulators are so low that they may not exist in all TCP4 complexes.

To affect TCP4 function, GI may also indirectly affect the DNA binding ability of TCP4 by regulating the stability of CDF1 and its homologues and changing the accessibility of the *CO* promoter. In LD, GI interacts with the F-box protein, FKF1 in a blue-light dependent manner and leads CDF1 for ubiquitination and proteasome-mediated degradation around dusk [15, 17]. CDF1 and its homologues function as strong *CO* repressors and their binding sites are closely localized to TCP binding sites [14, 15, 55]. Since the *gi* mutation stabilizes CDF1 and CDF2 throughout the day [14, 17], this may change the chromatin structure and inhibit the accessibility of the *CO* promoter. It is noteworthy that introducing the *gi* mutation to *cdf1 2 3 5* still reduced *CO* expression especially around dusk in LD, suggesting that *GI* works with additional *CO* activators, other than CDFs, to regulate *CO* expression in the dusk of LD [14]. Although the contributions of uncharacterized CDF homologs need to be examined, we propose that TCP4 and likely other class II CIN-TCPs are GI-associated proteins that induce *CO* expression in the late afternoon of LD.

### TCP4 integrates plant developmental signals into photoperiodic flowering

It is well known that TCP4 and other class II CIN-TCPs play critical roles in various plant developmental processes [44, 46, 47, 49, 51]. In this study, we demonstrated a novel function of class II CIN-TCP as a photoperiodic flowering regulator. In addition to direct activation of *CO* expression, *TCP4* may indirectly activate *FT* expression through unknown mechanisms. Loss of *tcp4* significantly reduced *FT* expression on ZT16 without having much of an effect on *CO* expression at dusk (Fig 4), suggesting that *FT* regulation by *TCP4* might be independent from the *CO*-*FT* pathway. A previous study suggested the involvement of the SQUAMOSA PROMOTER BINDING-LIKE (SPL) family in TCP4-mediated *FT* regulation. SPL3 and SPL9 are direct and indirect activators of *FT*, and both of them are targeted by miR156, whose expression declines in an age-dependent manner [82–84]. *SPL3* is downregulated in the *tcp4* mutant and *jaw-*D, plants that overexpress miR319a [35, 46, 51]. Furthermore, TCP4 is capable of binding to the TCP binding site (TGGTCC) located in the upstream region of *SPL3* promoter *in vitro* [51], implying the function of TCP4 as a direct activator of *SPL3*. In leaf development, SPL9 physically interacts with TCP4 and interferes with complex formation between TCP4 and CUP SHAPED COTYLEDON (CUC2) [45]. It is possible that TCP4 may enhance SPL9 stability through physical interaction, which causes *FT* accumulation. Given that TCP4 promotes onset of cell differentiation and leaf senescence [46, 51], TCP4 may promote flowering response by directly activating *CO* expression and potentially indirectly promoting *FT* expression as leaves mature during the course of plant development.

Our results indicated that TCP4 preferentially interacts with miR319-targeted CIN-TCPs and that they work together (Figs 4 and 8 and Table 1). Previous studies indicated that miR319 expression is induced by multiple stresses such as drought, salt or cold temperatures [85, 86]. In addition, overexpression of rice *miR319* confers salt tolerance in creeping bentgrass, *Agrostis stolonifera* [87]. Although the role of miR319 in stress response remains largely unknown, it is possible that the miR319-TCP pathway affects flowering in response to environmental stress. Therefore, our findings imply that TCP4 may connect plant development or possibly stress responses to the photoperiodic flowering pathway.

## Materials and methods

### Plant materials and growth conditions

The Colombia-0 (Col-0) accession was used as a wild type for all experiments. The *tcp3-1*, *tcp4-1*, *tcp5-1 tcp10-1*, *tcp13-2*, *fbh2-1* and *fbh3-1* mutant lines were described previously [16, 40]. The higher order *tcp* mutants (*tcp3-1 4–1 10–1* and *tcp3-1 tcp4-1 tcp5-1 tcp10-1 tcp13-2*: *tcp-Q*) were described previously [41]. To generate *TCP3*, *TCP4* and *TCP10* overexpressing transgenic plants, the coding regions of each *TCP* gene were amplified from cDNA derived from LD-grown wild-type plants using the following primers (sequences underlined are necessary for pENTR/D-TOPO cloning); 5′-CACCATGGCACCAGATAACGACCATTTC-3′ and 5′-TTAATGGCGAGAATCGGATGAAGC-3′ for *TCP3*, 5′-CACCATGTCTGACGACCAATTCCA-3′ and 5′-ATGGCGAGAAATAGAGGAAGC-3′ for *TCP4*, 5′-CACCATGGGACTTAAAGGATATAGCGTC-3′ and 5′-TTAGAGGTGTGAGTTTGGAGGAG-3′ for *TCP10*. The amplified *TCP* cDNAs were cloned into the pENTR/D-TOPO vector (Life technologies). After the sequences of the *TCP* genes were confirmed, each *TCP* gene was transferred, using Gateway LR clonase II (Life technologies), to pB7WG2 binary vector [88], which contains the CaMV 35S promoter-driven expression cassette. These constructs were introduced to *Agrobacterium* strain ABI, and then transformed to wild-type plants (Col-0) possessing the *pCO*:*GUS* reporter gene [9] using conventional floral-infiltration methods. The target site of microRNA319 /JAW (miR319/JAW) in the *TCP4* coding sequences was replaced with a non-target sequence by site-directed mutagenesis with the following primers (5′-CTCAGAGGGGTCCCTTGCAAAGTAGCTACAGTCCCATGATCCG-3′ and 5′-CGGATCATGGGACTGTAGCTACTTTGCAAGGGACCCCTCTGAG-3′) (designated as *mTCP4*). The coding region of *mTCP4* was amplified using the following primers (5′- CACCATGGCTTACCCATACGATGTTCCAGATTACGCTGCGATGTCTGACGACCAATTCCA-3′ and 5′-TCAATGGCGAGAAATAGAGGAAG-3′, the underlined sequences encode HA epitope tag) and cloned into the pENTR/D-TOPO vector (named *pENTR-HA-mTCP4*). To generate the *TCP4-3F6H* and *mTCP4-3F6H* construct, the coding regions of wild-type *TCP4* and *mTCP4* were amplified using the following primers (5′-CAGCCATGGCTGACGACCAATTCCATC-3′ and 5′-ATGGGATCCGATGGCGAGAAATAGAGGAAGC-3′), and inserted into the *Nco*I-*Bam*HI site of the pRTL2-3F6H vector designed for the in-frame fusion of the 3xFLAG-6xHis sequence to the 3′ region of a gene (named *pENTR-TCP4-3F6H* and *pENTR-mTCP4-3F6H*, respectively) [89]. To generate the *SUCROSE-PROTON SYMPORTER 2* (*SUC2*) promoter driven *mTCP4-3F6H* construct, 2.3 kb of the *SUC2* 5′ upstream promoter region was amplified using the following primers (5′-GGTGCATAATGATGGAACAAAGCAC-3′ and 5′-ATTTGACAAACCAAGAAAGTAAGAAAA-3′), and cloned into pRNTR5′-TOPO vector (Life Technologies). Both *SUC2* promoter and *mTCP4-3F6H* coding sequences were transferred to R4pGWB501 binary vector [90] using Gateway LR clonase II (Life technologies). The resultant plasmid *SUC2*:*mTCP4-3F6H* was introduced into Col-0. To make the *tcp fbh* septuple (*tcp3 tcp4 tcp10 fbh1 fbh 2 fbh 3 fbh4*) mutant line, the *fbh2-1 fbh3-1* double mutant and *tcp3-1 tcp4-1 tcp10-1* triple mutant lines were first crossed to make the *fbh2-1 fbh3-1 tcp3-1 tcp4-1 tcp10-1* quintuple mutant. Artificial microRNA (amiR) constructs of *FBH1* and *FBH4* [16] were tandemly fused and cloned into the pENTR/D-TOPO. The resultant cassette *amiR-FBH1-amiR-FBH4* was transferred into the pH7WG2 binary vector [88] to generate *35S*:*tandem amiR-FBH1-amiR-FBH4* by using Gateway LR clonase II. Finally, the construct was transformed into *fbh2-1 fbh3-1 tcp3-1 tcp4-1 tcp10-1* quintuple mutant lines. All homozygote lines were selected by several antibiotic- resistant markers and confirmed by genomic PCR. To generate *FBH1* overexpressing plants, a pENTR/D-TOPO vector harboring the coding region of *FBH1* [16] was transferred to the pK7WG2 or pH7WG2 vectors [88] to generate *35S*:*FBH1*, which was then transformed into both Col-0 harboring *SUC2*:*NTF* and *ACT2*:*BirA* [91], and the *tcp-Q* mutant [41]. To generate *FBH1* overexpressing lines in the *gi-2* mutant, *35S*:*FBH1 #24* [16] was crossed with the *gi-2* mutant. To generate the *35S*:*TCP4-3F6H* construct, *pENTR-TCP4-3F6H* was introduced into the pB7WG2 binary vector [88] to generate *35S*:*TCP4-3F6H*, which was then transformed into Col-0 harboring *CO*:*GUS* and the *gi-2* mutant. Transgenic plants were selected on culture media containing appropriate antibiotics, and all experiments were carried out using T_3_-T_4_ homozygous plants that have a single insertion of T-DNA.

### Flowering time experiment

For flowering time analysis, seeds were sown on the soil (Sunshine Mix #4; Sun Gro Horticulture) directly and stratified for 2–3 days at 4°C in darkness to synchronize the timing of germination. Plants were grown at 22°C in LD (16 h light/8 h dark) or SD (8 h light/16 h dark) conditions. Light was provided by full-spectrum white fluorescent light bulbs (F017/950/24” Octron; Osram Sylvania) with a fluence rate of 60–90 μmol m^-2^ s^-1^ in LD and 75–115 μmol m^-2^ s^-1^ in SD. Flowering time was measured by counting the number of rosette and cauline leaves on the main stem when they bolted. The experiments were repeated at least twice with at least 8 individual plants and similar results were obtained. The results are means ± standard errors of means (SEM).

### Yeast one-hybrid analysis

All reporter strains were generated in the yeast strain YM4271 according to manufacturer protocol (Clontech). 1.5 kb (1547 bp) and 500 bp of *CO* promoter fragments were amplified using the following primers (5′-CAGGTACCTGGGAAAGAGAAGTGCGGTGTAAGC-3′and 5′-CTCTCGAGAATAACTCAGATGTAGTAAGTTTG-3′ for 1.5 kb of the *CO* promoter, 5′-CACCGAGATACCTGAACAGTAATC-3′ and 5′-CTCTCGAGAATAACTCAGATGTAGTAAGTTTG-3′ for 500 bp of the *CO* promoter) and cloned into pENTR5′-TOPO vector (Life technologies), then transferred to pMW3 (pLacZi vector containing the gateway cassette [92]) following the manufacturer protocol (Life technologies). To generate mutated TCP binding *cis*-element reporter constructs, the TCP binding site GGACCAC (-263 to -257) located on the *CO* promoter was mutated to GGAACTC by PCR based site-directed mutagenesis. To generate translational fusions to the GAL4 activation domain (AD), the coding sequences of TCPs were amplified with the primers shown in Supplemental S4 Table, cloned in pENTR/D-TOPO, and subsequently transferred into pDEST22 (Life technologies). The pDEST22-MCS plasmid containing the pBluescriptII multi-cloning sites and *pDSET22-FBH3* [16] were used as the negative and positive control, respectively. Amplified sequences in all reporter and effector constructs were confirmed by sequencing. The transformation of *Arabidopsis* transcription factor library was performed in a 96-well format as previously described [93]. Transformation of AD constructs into the reporter strains and measurement of the β-galactosidase (β-gal) activity were performed in a 96-well format as previously described [94].

### Yeast two-hybrid analysis

To test protein-protein interactions in yeast, the cDNAs encoding full length of *TCP4* and *FBH1* were cloned into pENTR/D-TOPO (Life technologies) and then transferred to pASGW-attR bait vector through LR reaction [95]. The constructs for full-length GI, N-terminal half of GI (GI-N; amino acid residues 1–391), and C-terminal of GI (GI-C; amino acid residues 382–1173) and protocols for yeast two-hybrid assays were described previously [17].

### RNA preparation and gene expression analyses

Seedlings were grown on plates containing 1x Linsmaier and Skoog media (Caisson) and 3% sucrose in LD, SD for 10 days and harvested at 3 h intervals from 1 h after the onset of light (ZT1) to ZT22. To quantify the mRNA of genes involved in flowering regulation, total RNA was isolated from seedlings using illustra RNAspin Mini kit (GE Healthcare). To synthesize cDNA, 2 μg of total RNA was reverse-transcribed using iScript cDNA synthesis kit (Bio-Rad). cDNA was diluted to 5 times its volume with water, and 2 μl each of diluted cDNA was used for quantitative PCR (qPCR) analysis. The qPCR was performed in the buffer consisting of 1× Ex*Taq* buffer (Takara Bio USA), 0.1 mM Hepes-Na pH 7.5, 1× SYBR-Green (Molecular Probes), 10 nM fluorescein (Bio-Rad), 0.1% (w/v) tween-20, 5% (v/v) DMSO, 100 μg/mL BSA, 0.2 mM dNTPs, 250 nM primers and 1 U *Taq* DNA polymerase (New England Biolabs) using a MyiQ real-time detection system (Bio-Rad). The primers sequences used for amplification are shown in S4 Table. *ISOPENTENYL PYROPHOSPHATE / DIMETHYLALLYL PYROPHOSPHATE ISOMERASE* (*IPP2*) was used as an internal control for normalization. To amplify *CO*, *TCP3*, *TCP4*, and *FBH4*, we used the following 3-step PCR program: 1 min at 95°C, followed by 40–50 cycles of 10 sec at 95°C, 20 sec at 52–58°C, 20 sec at 72°C. To amplify the remaining genes, we used the following 2 step PCR program: 1 min at 95°C, followed by 40–50 cycles of 10 sec at 95°C, 20 sec at 60°C. Each data value shown in the figures is the mean values derived from three biological replicates and the value of each sample in each replicate is the average of values obtained from two technical replicated PCR reactions. Error bars indicate the SEM from three independent biological replicates.

### Analysis of tissue expressions (GUS staining)

For the construction of the *TCP4*:*GUS* reporter gene, the 5′ upstream region of the *TCP4* coding sequence (-3063 to -1, the translation initiation site was counted as +1) was amplified from Col-0 genomic DNA using the following primers (5′-CACCTGACTAAATGTTTAACCAACCAATG-3′ and 5′-TGGTAGAGCATATTCGTCGAGACG-3′) and cloned into the pENTR/D-TOPO vector, then transferred to the pGWB3 binary vector [96]. Transgenic lines carrying *TCP3*:*GUS*, *TCP5*:*GUS* and *TCP10*:*GUS* reporters were described previously [40]. Transgenic plants were grown in LD conditions for 12 days and fixed with cold 90% acetone on ice for 10–15 min. Subsequently, plants were incubated at 37°C in the staining buffer [0.5 mM X-Gluc, 50 mM sodium phosphate pH 7.2, 0.5 mM of K_4_Fe(CN)_6_ and 0.5 mM K_3_Fe(CN)_6_]. After staining, the samples were bleached and dehydrated with a sequence of buffers: 30% ethanol, fixing solution (50% ethanol, 5% acetic acid and 3.7% formaldehyde), 80%, and 100% ethanol for 30 min each. The staining patterns of GUS activity of more than 20 individual T_1_ transgenic plants were analyzed, and the data from the T_2_, T_3,_ and T _4_ population plants which showed the representative staining patterns of the T_1_ populations are shown.

### Chromatin immunoprecipitation assays

Approximately 1.5 to 2 g (fresh weight) of 10-day-old seedlings harvested at the indicated time point were ground into fine powder in liquid N_2,_ and then homogenized in 10 ml of the nuclei extraction buffer [0.4 M sucrose, 10 mM Tris-HCl pH 8.0, 10 mM MgCl_2_, 5 mM β-mercaptoethanol, 0.1 mM PMSF, 50 μM MG-132, 1mM Na_3_VO_4_, 1mM NaF, and Complete protease inhibitor cocktail tablets (Roche)]. Cross-linking reaction was performed by treating with 1% formaldehyde for 10 min at 4°C. The cross-linking reaction was stopped by adding glycine to a final concentration of 0.15 M and incubating at 4°C for 5 min. After filtration with miracloth, and centrifugation at 10,000 *g* at 4°C for 10 min, nuclei-containing pellets were washed twice in the nuclei wash buffer [0.25 M sucrose, 10 mM Tris-HCl pH 8.0, 10 mM MgCl_2,_ 1% (w/v) Triton X-100, 5 mM β-mercaptoethanol, 0.1 mM PMSF, 50 μM MG-132, 1mM Na_3_VO_4_, 1mM NaF, and Complete protease inhibitor cocktail tablets]. Isolated nuclei were lysed in the nuclei lysis buffer (50 mM Tris-HCl pH 8.0, 10 mM EDTA, 1% SDS, 1 mM PMSF, and Complete protease inhibitor cocktail tablets) and sonicated to shear DNA to an average size of 500 to 1,000 bp. The chromatin solution was diluted to 1 ml with ChIP dilution buffer (16.7 mM Tris-HCl pH 8.0, 167 mM NaCl, 1.1% Triton X-100, 1.2 mM EDTA, 0.1 mM PMSF, 50 μM MG-132, 1mM Na_3_VO_4_, 1mM NaF, and Complete protease inhibitor cocktail tablets). Immunoprecipitation was performed using Dynabeads Protein G (Life technologies). The beads were pretreated with anti-FLAG antibody (A8592, Sigma) and incubated with chromatin solution in an ultrasonic water-bath for 20 min, followed by 1.5-h incubation at 4°C. After washing with low salt buffer (20 mM Tris-HCl pH 8.0, 150 mM NaCl, 0.1% SDS, 1% Triton X-100, 2 mM EDTA), high salt buffer (20 mM Tris-HCl pH 8.0, 500 mM NaCl, 0.1% SDS, 1% Triton X-100, 2 mM EDTA) and TE buffer (10 mM Tris-HCl pH 8.0, 1 mM EDTA), immunocomplexes were eluted from beads, reverse cross-linked at 65°C overnight, and treated with proteinase K that digests all proteins. DNA was extracted and eluted in 140 μl of volume using QIAquick PCR purification kit (Qiagen), according to manufacturer protocol. 3μl aliquots were used for qPCR reaction. The forward and reverse primer pairs that were used to amplify the genome sequence designated as *CO* amplicon 1 to amplicon 7 are shown in S4 Table. The three-step PCR cycling program was used as follows: 1 min at 95°C, followed by 60 cycles of 10 sec at 95°C, melting temperatures (57°C for amplicons 1–4 and 60°C for amplicons 5–7 and *UBQ*) for 20 sec, and 72°C extension for 15 sec. The immunoprecipitation efficiency (%) against the total input was calculated for each amplicon using the following formula: 0.02×2^(Ct input-Ct ChIP)^ ×100.

### Transient luciferase reporter assays and co-immunoprecipitation assays in *Nicotiana benthamiana*

To amplify the *CO* promoter sequence, we used different combinations of forward primers [for -1 kbp_F (5′-CACCTACAAGTGTCGTTTGTATTAG-3′), -500bps_F (5′-CACCTCTAACCTTTGTATAGGTAGT-3′)] with reverse primer (5′-AAAGCTTATATCTGGTGTGAGAGA-3′). Amplified PCR products were cloned into pENTR/D-TOPO vector. *CO* promoter fragments were transferred to firefly luciferase (Luc) vector pFLASH (Gateway compatible version of pPZPXomegaLUC^+^ [97]) to generate reporter plasmids by using LR clonase II. To generate effector plasmids, *pENTR-HA-mTCP4* was transferred to pB7WG2 vector [88] to generate *35S*:*HA-mTCP4* by using LR clonase II plus. *35S*:*GFP* (used as negative control of effector plasmids) and *35S*:*Renilla Luc* (RLuc, used as internal control to measure transient expression efficiency) were described previously [98]. *N*. *benthamiana* samples were harvested three days after the transfection. The expression levels of HA-mTCP4, GFP, and RLuc were analyzed by western blot using a HRP-conjugated anti-HA antibody (3F10, Roche), a HRP-conjugated anti-GFP antibody (ab6673, Abcam), and an anti-Renilla Luc antibody (PA532210, Life technologies), respectively. Total protein was extracted using 2x Laemmli sample buffer, and the same amount of total protein was loaded to each lane. Luc reporter activity was analyzed using the Dual-Luciferase Assay System (Promega) based on manufacturer instructions. Soluble proteins were extracted with Passive Lysis Buffer (Promega) supplemented by Complete Protease Inhibitor Mixture tablets (Roche). The luminescence of firefly LUC and Renilla LUC were analyzed using a Victor3 V multiwall plate reader (Perkin-Elmer).

To generate *GI* overexpression construct for transient co-IP assays, the full length of *GI* cDNA without the stop codon was amplified and inserted into the pENTR/D-TOPO vector. After sequences were verified, the *GI* cDNA was transferred into the pB7HFc vector harboring 6xHis-3xFLAG (6H3F) sequences designed for in-frame fusion to the 3′ region of a gene [99] by LR reaction. *35S*:*HA-mTCP4* and *35S*:*GI-6H3F* constructs were infiltrated into approximately 3-week-old *N*. *benthamiana* plants grown in LD conditions and subjected to co-IP experiments as previously described [67].

### BiFC assay in *N*. *benthamiana* leaf epidermal cells

To generate binary vectors for the BiFC assay, the coding sequences of *CIN-TCP*s, *FBH1* [16], and *GI* [17] were amplified with the primers shown in S4 Table and cloned in pENTR/D-TOPO, and subsequently transferred into pSITE-3C1/N1 binary vectors to generate in-frame fusion of either the N-terminal or C-terminal half of EYFP [100]. The nuclear-localized form of GST-tag (GST_NLS_), which was generated from the plasmid pGEX-4T1, was used as the negative control. H2B-RFP was used for the nuclear marker [98]. Approximately 20-day-old *N*. *benthamiana* plants grown in LD at 22°C were used for agroinfiltration. The *Agrobacterium* strain GV3130 containing plasmids of interest was grown to the stationary phase. Bacterial cells were harvested by centrifugation and resuspended to OD_600_ of 0.4 in MES buffer [10 mM MgCl_2_, 10 mM MES pH 5.6, 150 μM acetosyringone (Sigma)]. After 4 h of incubation at room temperature in MES buffer, *Agrobacterium* solution was infiltrated into the abaxial air spaces of leaf tissue using 1-mL syringes. Two to three days after infiltration, YFP and RFP images of the tissue were analyzed with a confocal laser scanning microscope (TCS SP5; Leica Microsystems). Approximately 50 to 100 cells that express H2B-RFP were observed in each combination. We called positive interactions when more than 70% of the H2B-RFP positive cells showed reconstituted specific YFP signals, whereas no signals were observed from the combinations with GST_NLS_.

### Protein extraction and immunoblot analysis

Total protein was extracted from seedlings grown under LD and harvested at 4h intervals from ZT0 to ZT20 on day 10. Whole protein extract was extracted using a buffer containing 50 mM Na-phosphate pH 7.4, 100 mM NaCl, 10% (v/v) glycerol, 5 mM EDTA, 1 mM DTT, 0.1% Triton X-100, 50 μM MG-132, 2mM Na_3_VO_4_, 2 mM NaF, and Pierce Protease Inhibitor Tablets, EDTA-free. Approximately 30 μg protein in each sample was run in 12% SDS-PAGE gels, and transferred to Nitrocellulose membranes (Bio-Rad). TCP4-3F6H proteins were detected by using a HRP-conjugated anti-FLAG antibody (A8592, Sigma), whereas histone H3 protein was detected by anti-histone H3 antibody (ab1791, Abcam), followed by a HRP-conjugated goat anti-rabbit antibody (Thermo Fisher Scientific). Immunoreactive proteins were visualized with SuperSignal West Pico Chesmiluminescent Substrate (Thermo Fisher Scientific) and Amersham ECL Select Western Blotting Detection Reagent (GE healthcare). For protein quantification, signals from immunoblottted membranes incubated in chemiluminescent detection reagents were imaged and quantified by a high sensitivity cooled CCD camera system (NightOWL, Berthold) and the IndiGo program (Berthold). Histone H3 was used for normalization of a protein in whole extract.

### Tandem affinity purification coupled mass spectrometry (TAP-MS) analysis

TAP-MS analysis using functional TCP4-3F6H protein was performed based on the previously described methods with slight modification [67]. Approximately 10 g of *35S*:*TCP4-3F6H* lines in both WT and *gi-2* background were harvested on ZT13 of day 14. To exclude nonspecific binding, Col-0 seedlings without any transgenes were used as a control. Samples were ground into fine powder in liquid nitrogen and resuspended in SII buffer [100 mM Na-P buffer pH 7.4, 150 mM KCl, 5 mM EDTA, 5mM EGTA, 1% Triton X-100, 0.1% Sodium deoxycholate, Complete Protease Inhibitor Mixture Tablet, EDTA-free (Roche), PhosStop Phosphatase Inhibitor Mixture (Roche), 100 μM PMSF, and 50 μM MG-132]. Samples were sonicated and filtered twice through 0.45-μm pore size PVDF membrane (Millipore), followed by centrifugation to remove cell debris. The clear supernatant was incubated with anti-FLAG antibody (A8592, Sigma)-bound Dynabeads (Invitrogen). Once the TCP4-3F6H proteins were captured by the Dynabeads, the beads were washed three times with SII buffer with 0.1% Triton, and without the deoxycholate, protease and phosphate inhibitor mixtures. The rest of the purification steps were same as previously described [67].

On-bead digestion for TCP4-interacting proteins was performed using the following methods. Disulfide bonds of proteins bound to Dynabeads were reduced using 50 μl of 0.2% PPS surfactant (Expedeon) in 100 mM ammonium bicarbonate with 1 μl 500 mM Tris (2-carboxyethyl) phosphine hydrochloride (Thermo Fisher Scientific) for one hour at 60°C. The sample was cooled to room temperature and the cysteine thiols alkylated by the addition of 1.1 μl of 500 mM iodoacetamide for 20 min. The beads were then digested with the addition of 1 μg trypsin overnight at 37°C with constant agitation. The supernatant containing the tryptic peptides were removed, and combined with a methanol wash of the beads. The combined supernatants were dried using vacuum centrifugation, and resuspended in 15 μl of 0.1% trifluoroacetic acid prior to LCMS analysis.

All mass spectrometry was performed on a LTQ-FT (Thermo Fisher Scientific). Three microliters of sample digest were loaded from the autosampler onto a 150-μm Kasil fritted trap packed with 3 u Dr. Maisch ReproSil-Pur C18-AQ beads to a bed length of 2 cm at a flow rate of 2 μl/min. After loading, the trap was brought on-line with a pulled fused-silica capillary tip (75-μm i.d.) also packed with the same Dr. Maisch beads mounted in an in-house constructed microspray source and placed in line with a Waters Nanoacquity binary UPLC pump plus autosampler. Peptides were eluted off the column using a gradient of 2–35% acetonitrile in 0.1% formic acid over 60 min, followed by 35–60% acetonitrile over 5 min at a flow rate of 250 μl/min. The mass spectrometer was operated using data dependent acquisition (DDA) where a maximum of seven MS/MS spectra were acquired per MS spectrum. The resolution for MS was 100,000 at *m/z* 400, and for MS/MS the linear ion trap provided unit resolution. The automatic gain control targets for MS in the FT was 1×10^6^, whereas for MS/MS it was 8000, and the maximum fill times were 20 and 80 ms, respectively. The MS/MS spectra were acquired using an isolation width of 2 *m*/*z* and a normalized collision energy (NCE) of 35. MS/MS acquisitions were prevented for precursor charge states of 1, or if the charge state could not be discerned from the MS spectrum. Dynamic exclusion (including all isotope peaks) was set for 30 sec.

Peptide identification was performed using Comet, searching against a Uniprot Arabidopsis protein sequence database, and using Percolator with a *q*-value cutoff of 0.01. Cysteine residue masses were considered statically modified by iodoacetamide, and methionine dynamically modified by a single oxidation. Precursor mass tolerance was 10 ppm, and product ion tolerance 0.5 Da. The principle of parsimony was used for protein inference. Spectral counts per protein were then analyzed using the Fisher’s Exact test, where alpha was set at 0.01 and multiple hypothesis correction was carried out using the Bonferroni correction. Frequently identified contaminant proteins were subtracted based on previous reports [101].

### Statistical analysis

Statistical analyses were done using R Statistical Computing software (v3.2.3; R Core Team, 2015). The statistical significance in yeast one hybrid analyses, transient assays in *N*. *benthamiana*, and ChIP analyses was determined using Welch’s *t*-test or Student’s *t*-test. The statistical significance in gene expression analyses was determined by one-way ANOVA with strains as main effects. Pairwise comparisons were determined using Dunnet’s test on each time point. Student’s *t*-test was used to determine the statistical difference in gene expression or protein expression between two strains on each time point. The statistical significance in flowering time measurement was determined by Tukey’s honestly significant difference test. The *p* values are indicated in the figure legends.

## Supporting information

S1 Fig Gene expression patterns in the *35S:TCP4* and *35S:TCP3* lines under different photoperiod conditions. (A to D) Results of qPCR analysis that show gene expression patterns of *TCP4* (A and B), *TCP3* (C and D), *CO* (E and G) and *FT* (F and H) in LD (A and C) and SD (B, D, and E to H) in *35S*:*TCP4* (A,B, E and F), *35S*:*TCP3* (C, D, G and H), and wild type (WT) plants. Time indicates hours (h) after light onset within a day. Bars above the traces represent light conditions; open and filled bars represent day and night, respectively. All expression data were normalized against *IPP2* and shown relative to the average expression of each gene in WT. *FT* expression values in SD are shown relative to the WT average expression value in LD shown in Fig 2. Significant differences from WT values are indicated by asterisks (*p<*0.05, Dunnett’s test). Data represent means ± SEM (*n* = 3). (I and J) Results of qPCR analysis that show the expression level of *TCP10* (I) and *CO* (J) in *35S*:*TCP10* lines and WT harvested at ZT16 in LD. Significant differences are indicated by asterisks (HSD test; *p<0.05, **p<0.01). Data represent means ± SEM (*n* = 3).
(EPS)

S2 Fig Gene expression patterns and protein profiles in the *SUC2:mTCP4-3F6H* plants. (A and B) Results of qPCR analysis that show the gene expression pattern of *TCP4* (A) and *CO* (B) in LD. Significant differences from WT are indicated by asterisks (*p<*0.05, Student’s *t*-test). Data represent means ± SEM (*n* = 3). (C) The representative protein profiles of mTCP4-3F6H in LD. Ponceau S-staining of RbcL is shown as the loading control. Similar results were obtained from 3 independent experiments.
(EPS)

S3 Fig Flowering phenotypes and gene expression patterns in higher order *tcp* mutants in SD. (A) Flowering phenotypes of higher order *tcp* mutants in SD. Total number of rosette leaves and cauline leaves generated from the main stem were counted when plants bolted. ns: no significance (HSD test). Data represent means ± SEM (*n*≥16). (B and C) Results of qPCR analysis that shows gene expression patterns of *CO* (B) and *FT* (C) in SD in higher order *tcp* mutants. *FT* expression values in SD are shown relative to the WT average expression value in LD shown in Fig 4. Significant differences from WT values are indicated by asterisks (*p<*0.05, Dunnett’s test). Data represent means ± SEM (*n* = 3).
(EPS)

S4 Fig Gene expression patterns in *tcp fbh* multiple mutants. (A and B) Results of qPCR analysis that shows gene expression patterns of *FBH1* (A) and *FBH4* (B) in LD. Significant differences from WT are indicated by asterisks (Dunnett’s test, *p<*0.05). Data represent means ± SEM (*n* = 3).
(EPS)

S5 Fig Gene expression patterns and flowering phenotype in the *35S:FBH1* lines in LD. (A to C) Results of qPCR analysis that show gene expression patterns of *FBH1* (A), *CO* (B) and *FT* (C) in the *35S*:*FBH1*, WT, and *tcp-Q* plants grown in LD. The data set shown in Fig 5 was used for WT, *35S*:*FBH1 #9*, and *tcp-Q*. Significant differences between the *35S*:*FBH1* lines and their background strains are indicated by asterisks (*p<*0.05, Student’s *t*-test). Data represent means ± SEM (*n* = 3).
(EPS)

S6 Fig Analysis of protein-protein interaction between FBH1 and GI and their genetic interaction. (A) Results of the yeast two-hybrid assay between FBH1 and GI. The full-length of FBH1 and full-length or truncated GI fused to either the DNA-binding domain (DBD) or the activation domain (AD) of Gal4 were tested under selective (–LWH, top) and non-selective (–LW, bottom) conditions. For GI, N and C indicate the amino acid residues1-391 and 382–1173, respectively. (B) BiFC assays of interaction between FBH1 and GI. The full-length of GI fused to the N-terminal half of enhanced YFP (GI-YFP^n^) and the full-length of FBH1 fused to the C-terminal half of enhanced YFP (FBH1-YFP^c^) were expressed in *N*. *benthamiana* leaf epidermal cells. H2B-RFP was used for the nuclear marker. Images from YFP and RFP channels were merged with bright-field (BF) images. Nuclear-localized form of GST fragments (GST_NLS_) fused to YFP^n^ or YFP^c^ were used as negative controls. Scale bar, 20 μm. (C and E to H) Results of qPCR analysis that show gene expression patterns of *FBH1* (C), *CO* (E and F), *FT* (G), and *SOC1* (H) in *35S*:*FBH1*, WT, and *gi-2* grown in LD. *CO* expression profiles shown in (E) were enlarged in (F) to show the difference in lower values within the lines. Significant differences between the *35S*:*FBH1* lines and their background strains are indicated by asterisks (Student’s *t*-test, *p<*0.05). Data represent means ± SEM (*n* = 3). (D) Flowering phenotype of the *35S*:*FBH1* plants in LD. Total number of rosette leaves and cauline leaves generated from the main stem were counted when plants bolted. Significant differences are indicated by asterisks (HSD test, *** *p<*0.001). Data represent means ± SEM (*n* = 16).
(EPS)

S7 Fig Gene expression patterns and protein profiles in the *35S:TCP4-3F6H* lines in LD. (A to C) Results of qPCR analysis that show gene expression patterns of *TCP4* (A), *CO* (B), and *FT* (C) in the *35S*:*TCP4-3F6H*, WT, and *gi-2* plants grown in LD. The same data set for WT and *gi-2* shown in Fig 7 was used. Significant differences between the *35S*:*TCP4-3F6H* lines and their background strains are indicated by asterisks (Student’s *t*-test, *p<*0.05). Data represent means ± SEM (*n* = 3). (D) The representative protein profiles of TCP4-3F6H in WT and *gi-2* grown in LD are shown. Histone H3 protein was used as the loading control. The numbers above the images indicate time (h) after light onset within a day. (E) The quantified results of the TCP4-3F6H protein profiles shown in (D) were obtained from 3 independent biological replicates. Significant differences are indicated by asterisks (Student’s *t*-test, *p<*0.05). Data represent means ± SEM (*n* = 3).
(EPS)

S8 Fig Results of BiFC assays of interactions among class II TCP proteins. Full length of TCP2, 3, 5, 10, 13, 17, and 24 fused to YFP^c^ were co-overexpressed with GST protein fused to YFP^n^ in *N*. *benthamiana* leaf epidermal cells. H2B-RFP was used for a nuclear marker. Images from YFP and RFP channels were merged with bright-field (BF) images. Scale bar, 20 μm.
(EPS)

S9 Fig Posttranslational modifications of TCP4 identified by TAP-MS analysis. The peptide coverages for TCP4 protein identified by MS analysis are indicated. The TCP4 peptides identified by MS analysis are highlighted in red. The percent coverage was 73%. The number of peptides recovered is shown in Table 1 and S2 Table. Phosphorylated and ubiquitinated amino acid residues are indicated by light green and light blue boxes, respectively. The putative CKII phosphorylation site is underlined.
(EPS)

S10 Fig ChIP assays in the *35S:TCP4-3F6H* lines at ZT13. Results of ChIP analysis using *35S*:*TCP4-3F6H* in either WT and *gi-2* backgrounds harvested on ZT13 are shown. Amplicons located in the *CO* promoter are described in Fig 3D. Significant differences are indicated by asterisks (Student’s *t*-test, *p<*0.05). Data represent means ± SEM (*n* = 5).
(EPS)

S1 Table Results of the large-scale yeast one-hybrid analysis using the *CO* promoter. (XLSX)

S2 Table List of TCP4 interacting proteins in WT and *gi-2* backgrounds identified by the TAP-MS analysis. (XLSX)

S3 Table List of phosphorylated and ubiquitinated residues identified within TCP4 peptide sequences. (XLSX)

S4 Table Primers used in this study. (XLSX)
